# Simultaneous Determination of Water- and Fat-Soluble Arsenic Species by HPLC-ICP-MS in Food Samples: A Pilot Study

**DOI:** 10.3390/foods15132304

**Published:** 2026-06-28

**Authors:** Dorota Jakkielska, Joanna Wolska, Marcin Frankowski, Anetta Zioła-Frankowska

**Affiliations:** Faculty of Chemistry, Adam Mickiewicz University, Uniwersytetu Poznańskiego 8, 61-614 Poznan, Poland; dorota.jakkielska@amu.edu.pl (D.J.); j.wolska@amu.edu.pl (J.W.); marcin.frankowski@amu.edu.pl (M.F.)

**Keywords:** food, arsenic forms, chromatography, HPLC-ICP-MS, algae, fish

## Abstract

Arsenic is a highly toxic element that occurs naturally and widely in the environment. Its toxicity depends on the forms in which it occurs, with inorganic arsenic species considered more toxic than organic ones. However, besides the commonly analyzed arsenic species [As(III), As(V), AsB, DMA, and MMA], there are also fat-soluble organic species, arsenolipids, which can be as toxic as or even more toxic than inorganic species. Therefore, to accurately assess the health risks resulting from the consumption of foods containing arsenic, a speciation analysis is needed. Taking this into account, the study aimed to develop a method for the simultaneous determination of both water-soluble [As(III), As(V), DMA, and AsB] and fat-soluble (AsHC 360 and AsFA 362) arsenic species using HPLC-ICP-MS. The present study reports the results of a preliminary optimization investigation. The method development and analyses were conducted qualitatively using standards and food samples—algae, salmon and tuna. The developed method allowed for the full separation of arsenolipids and the partial separation of water-soluble arsenic species in a single run. As there are no commercially available arsenolipid standards, the syntheses of AsHC 360 and AsFA 362 were also a part of the study. Additionally, the synthesized arsenolipids were analyzed by LC-MS.

## 1. Introduction

Arsenic is a highly toxic element that occurs naturally and widely in the environment. Its sources may be either natural (biogeochemical processes, such as erosion of minerals and rocks exposed to water or air) or anthropogenic (human activities, such as mining and metal refining; use of fertilizers, insecticides and pesticides; and industrial waste) [1,2,3]. Arsenic is considered to be carcinogenic to humans. It is important to note that it exists in various forms that differ in their chemical and physical properties and toxicity [4]. Inorganic arsenic species are believed to be more toxic than organic species, and their toxicity decreases in the following order: arsenite, As(III) > arsenate, As(V) > monomethyl arsenic, MMA > dimethyl arsenic, DMA > arsenocholine, AsC > arsenobetaine, AsB, with AsC and AsB considered nontoxic [5,6]. Exposure to arsenic can lead to a variety of health issues, including cancers of the bladder, kidney, liver, lung, prostate and skin, as well as damage to skin and cardiovascular, endocrine, immune, nervous, reproductive and respiratory systems. It may also contribute to neurodegenerative diseases [4,5,7,8]. The toxicity of arsenic and the differences in toxicity among its various forms highlight the importance of speciation analysis. Unlike the determination of total arsenic content alone, speciation analysis provides essential information for assessing the potential health risks associated with arsenic exposure. Generally, arsenic speciation analysis is performed for rice and rice products [9,10,11,12,13,14,15,16,17,18,19,20,21,22,23], fish, seafood and other marine products [1,9,10,18,19,24,25,26,27,28,29,30,31,32,33,34,35,36], although various other food products have also been analyzed, including cereal products [17,37], wines and fruit juices [38,39], mushrooms [40,41], vegetables [15,18], chicken [42,43] and dietary supplements intended for children and pregnant women [44]. Rice and marine products, such as fish and seafood, are among the major sources of arsenic in the human diet. Moreover, marine products and algae, including seaweed, are the primary focus of arsenolipid analysis [45,46,47,48,49,50,51,52,53,54,55].

Arsenolipids are compounds that contain arsenic and are soluble in fats. They have been determined in a variety of marine products and can be classified into categories such as arsenic-containing hydrocarbons (AsHCs), arsenic-containing fatty acids (AsFAs) and arsenic-containing phospholipids (AsPLs) [56,57,58]. The toxicity of arsenolipids can be comparable to, or even greater than, that of inorganic arsenic species [7,57,59,60,61,62]. Importantly, arsenolipids have been detected in the milk of nursing mothers [6,63] and in the developing brains of pilot whales [56], indicating that even the youngest and most vulnerable individuals may be exposed to their toxic effects.

Analysis of arsenolipids has been performed using reverse-phase high-performance liquid chromatography (HPLC) with inductively coupled plasma mass spectrometry (ICP-MS) and electrospray ionization mass spectrometry (ESI-MS), often run simultaneously by splitting the eluant between ICP-MS and ESI-MS [64]. The use of both mass spectrometers enabled not only the separation and determination of arsenolipids, but also the identification of unknown arsenolipids, which would otherwise be hindered by species coelution [65] and their degradation in the plasma source [50,66]. Generally, columns functionalized with octyl (C8) or octadecyl (C18) groups were used, and the analyses were performed under gradient elution conditions using a water-based mobile phase A and a methanol- or ethanol-based mobile phase B. Both mobile phases typically contained acetic acid, formic acid or ammonium acetate [45,48,49,50,51,64,67,68,69,70] (Table S1). However, there are currently no certified reference materials (CRMs) certified for arsenolipids and no commercially available arsenolipid standards. It makes their analysis, as well as ensuring the quality and accuracy of the obtained data and the reproducibility of the applied methods, particularly challenging [48,66,71,72]. Syntheses of arsenolipids with high yields are difficult [66]; nevertheless, the syntheses of selected arsenolipids have been successfully described in several published studies [64,73,74,75,76,77].

Analysis of water-soluble arsenic species, most commonly As(III), As(V), DMA and MMA, but also AsB and AsC, and less frequently TETRA and TMAO, has generally been carried out using HPLC-ICP-MS and water-based mobile phases under either gradient or isocratic elution conditions. These mobile phases consisted of ammonium salts (ammonium acetate, ammonium bicarbonate, ammonium carbonate, ammonium formate, ammonium nitrate, ammonium oxalate, ammonium phosphate, diammonium phosphate), monopotassium phosphate, malonic acid or pyridine. The analyses employed anion-exchange columns and/or cation-exchange columns [9,11,12,14,18,19,27,30,32,36,41,69,70,78,79,80,81,82,83,84,85,86,87] (Table S2). Overall, the comparison of the analytical methods reported for HPLC-ICP-MS analyses of fat-soluble and water-soluble arsenic species indicates that ammonium acetate has been used as the mobile phase component in the analyses of both groups of compounds, suggesting its potential suitability for their simultaneous analysis.

Therefore, the aim of the study was first to synthesize selected arsenolipids, AsFA 362 [15-(dimethylarsinyl)pentadecanoic acid] and AsHC 360 (1-(dimethylarsinyl)heptadecane), following previously published procedures. These arsenolipids were selected based on their reported toxicity and published studies confirming their occurrence in the environment. Then, using the synthesized arsenolipids, the study sought to improve arsenic speciation analysis, enabling a more accurate assessment of the environmental contamination and the health risks associated with the consumption of arsenic-containing foods, by developing a method for the simultaneous speciation analysis of water-soluble species [As(III), As(V), AsB and DMA] and arsenolipids (AsFA 362 and AsHC 360). The analyses were conducted qualitatively using mostly standards; however, food samples (algae, salmon and tuna) were also analyzed. It should be emphasized that, as noted above, no commercially available arsenolipid standards currently exist. Therefore, their successful synthesis was essential for the study.

## 2. Materials and Equipment

### 2.1. Reagents Used for Synthesis and Analysis

The syntheses of AsFA 362 and AsHC 360 were carried out using the following reagents: 1-bromoheptadecane (≥95.0%, Merck, Buchs, Switzerland), 15-bromopentadecanoic acid (98%, AmBeed, Buffalo Grove, IL, USA), cacodylic acid (DMA, Sigma–Aldrich, St. Louis, MO, USA), chloroform (≥99.8%, Honeywell, Charlotte, NC, USA), diethyl ether (Supelco, Seelze, Germany), ethanol (99.9%, STANLAB, Warsaw, Poland), ethyl acetate (Carlo Erba, Paris, France), HCl (≥37%, Sigma–Aldrich, Vienna, Austria), NaOH (Supelco, Germany), potassium iodide (≥99%, Sigma–Aldrich, Bangalore, India), sodium bisulfite (Sigma–Aldrich, India) and sodium sulfate (Sigma–Aldrich, Seelze, Germany). The mobile phases (HPLC-ICP-MS) were prepared using ammonium acetate (≥96.0%, Merck, Darmstadt, Germany), ammonium nitrate (Sigma–Aldrich, St. Louis, MO, USA) and methanol (Honeywell, Paris, France) and their pH was adjusted with acetic acid (≥99.8%, Sigma–Aldrich, Germany) and ammonia (Sigma–Aldrich, St. Louis, MO, USA). The mobile phase (LC-MS) was prepared using acetonitrile (hypergrade for LC-MS, Merck, LiChrosolv^®^, Germany), water (for chromatography, LC-MS grade, Merck, LiChrosolv^®^, Germany) and formic acid (98–100%, for LC-MS, Merck, LiChropur, Germany). The standard solutions of the water-soluble arsenic species were prepared using arsenic(III) standard for ICP (Sigma–Aldrich, Sankt Gallen, Switzerland), arsenic(V) standard for ICP (Sigma–Aldrich, Switzerland), arsenobetaine (Sigma–Aldrich, St. Louis, MO, USA) and sodium cacodylate trihydrate (sodium salt of DMA(V), Sigma–Aldrich, St. Louis, MO, USA). The standards and samples were prepared using methanol (Honeywell, France). Ultrapure, deionized water was obtained with the Milli-Q Direct 8 purification unit (Merck, Millipore, Burlington, MA, USA).

### 2.2. Synthesis

**Figure 1:**
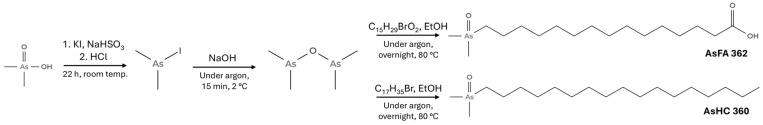
Synthetic schemes for AsFA 362 and AsHC 360.

The syntheses were conducted based on the procedures described by Arroyo-Abad et al. [74] and Taleshi et al. [64], with modifications introduced to accommodate the available laboratory equipment and experimental capabilities. The comparison of the synthetic procedures is presented in Tables S3–S5, while the corresponding synthetic schemes are shown in Figure 1. Among the most notable modifications, we extended the synthesis time of iododimethylarsine to 22 h, overnight, instead of using the shortened 4 h procedure reported by Arroyo-Abad et al. [74]. This adjustment facilitated the scheduling of the reaction and subsequent purification steps, including extraction and distillation. Additionally, we extracted the entire reaction mixture rather than only the dark brown bottom layer formed during the reaction, and we implemented a distillation step to obtain iododimethylarsine as a clear yellow oil. The complete synthesis procedures are described in the Supplementary Materials.

### 2.3. Equipment

#### 2.3.1. LC-MS

The synthesized arsenolipids, AsHC 360 and AsFA 362, were analyzed using an LC-MS/MS 8050 system (Shimadzu, Kyoto, Japan) operated in the scan (SCAN) and selected ion monitoring (SIM) modes. The arsenolipid samples, prepared in methanol, were introduced into the ESI source using a SIL 30AC autosampler (Shimadzu, Kyoto, Japan) with an injection volume of 1 µL. The mobile phase consisted of H_2_O/ACN (30/70) containing 1% formic acid. The ESI source parameters were as follows: nebulizing gas flow rate: 3 L min^−1^, heating gas flow rate: 10 L min^−1^, drying gas flow rate: 10 L min^−1^, interface temperature: 300 °C, DL temperature: 250 °C, heat block temperature: 400 °C. Additionally, the SIM analysis was followed by product ion scanning to exclude sodium adducts. If an ion was identified as an M+Na adduct, it was reassigned as M+H to improve the clarity of the presented results.

#### 2.3.2. HPLC-ICP-MS

The analysis of AsFA 362 and AsHC 360 was performed using a Prominence Inert HPLC system (Shimadzu, Kyoto, Japan) equipped with binary pumps (LC 20Ai) (Shimadzu, Kyoto, Japan), a vacuum degasser (DGU20A3R) (Shimadzu, Kyoto, Japan), a heated column compartment (CTO 20AC) (Shimadzu, Kyoto, Japan), an autosampler (SIL 20AC) (Shimadzu, Kyoto, Japan) and a system controller (CBM 20A) (Shimadzu, Kyoto, Japan) coupled to an ICPMS-2050 (Shimadzu, Kyoto, Japan). To reduce the volume of the organic solvent entering the plasma and to prevent the plasma instability or extinguishing due to the high methanol content [88], the column effluent was diluted with 1% HNO_3_ between the column and the nebulizer using an additional pump (Perimax 12) (Spetec GmbH, Erding, Germany). The operating parameters and details of the HPLC-ICP-MS method used for the analysis of the synthesized arsenolipids (AsFA 362 and AsHC 360, prepared in methanol) and for the analysis of the fish and algae samples are presented in Table 1.

During the development of a speciation analysis method for the synthesized arsenolipids (AsFA 362 and AsHC 360) simultaneously with the water-soluble arsenic species [As(III), As(V), DMA and AsB], various analytical columns and their combinations were evaluated (Table S6). The investigated columns included anion-exchange columns [Thermo Scientific Dionex IonPac AS22 (Thermo Scientific, Sunnyvale, CA, USA), Hamilton PRP-X110 (Hamilton, Bonaduz, Switzerland)], a mixed-mode cation- and anion-exchange column [Thermo Scientific Dionex IonPac CG5A (Thermo Scientific, Sunnyvale, CA, USA)], a C8 column [Shimadzu Shim-pack Scepter C8-120 (Shimadzu, Kyoto, Japan)] and several C18 columns [Supelco SUPELCOSIL LC-18 (Supelco, Bellefonte, PA, USA), Thermo Scientific Hypersil Duet C18/SAX (Thermo Scientific, Sunnyvale, CA, USA), Phenomenex PhenoSphere NEXT C18 (Phenomenex, CA, USA), Phenomenex HyperClone ODS (C18) (Phenomenex, CA, USA), Shimadzu Shim-pack Scepter C18-120 (Shimadzu, Kyoto, Japan)]. The mobile phase systems were based on a combination of a water-based phase A (1–20 mmol L^−1^ ammonium acetate at pH = 6.0–9.2 or 75 mmol L^−1^ ammonium nitrate at pH = 9.0) and a methanol-based phase B (1–20 mmol L^−1^ ammonium acetate at pH = 6.0–9.2), applied under various gradient programs (Table S6). The columns, mobile phases, pH values and gradient program parameters were selected based on the data reported in the scientific literature, as summarized in the Supplementary Materials (Tables S1 and S2). During the method development, the mobile phase flow rate was adjusted from an initial 1.0 mL min^−1^ (with most of the effluent directed to waste and only a small fraction introduced into the ICP-MS) to 0.2 mL min^−1^ and was later increased to 0.4 mL min^−1^. The post-column dilution flow rate was set at 0.6 mL min^−1^ and was later increased to 0.7 mL min^−1^.

### 2.4. Standard Solutions Preparation

To prepare the standard solutions of AsFA 362 and AsHC 360, a portion of crystallized arsenolipid was weighed and dissolved in 1 mL of methanol, from which a 10 mg L^−1^ stock solution was prepared in methanol. Working standards of lower concentrations, used for the analyses, were prepared by diluting the 10 mg L^−1^ stock solution with methanol. The standard solutions of the water-soluble arsenic species were prepared at a concentration of 10 mg L^−1^ in the water-based mobile phase A (10 mmol L^−1^ NH_4_OAc in water, pH = 6.0) and were diluted with the mobile phase A prior to analysis. For the mixed standard solutions, containing both arsenolipids and water-soluble arsenic species, methanol was used as the solvent. All the prepared standard solutions were stored in a refrigerator.

### 2.5. Samples and Their Preparation

Fresh salmon and tuna samples were purchased from stores in Poland. Their total arsenic content was determined and reported [89]. Following the previously described extraction procedure [90,91], the fish samples were frozen, lyophilized and homogenized. Subsequently, 200 mg of each sample was weighed into a polypropylene tube and 5 mL of MeOH/H_2_O (50:50, *v*/*v*) was added. The mixture was vortexed (60 s), heated and mixed in a thermoshaker (90 °C, 40 min, 300 rpm), and centrifuged (10 min, 9000 rpm). The resulting extracts were then analyzed by HPLC-ICP-MS. The methanol and water mixture was used with the intention of extracting both arsenolipids, which are soluble in methanol, and water-soluble arsenic species. Tibon et al. [91] reported that the use of aqueous methanol (50:50, *v*/*v*) resulted in high extraction recoveries, exceeding 90% in most samples. During the centrifugation of the salmon samples, an additional orange oil layer was formed. It was collected and analyzed separately after dissolving in 1 mL of methanol. The algae samples, including marine algae, red algae and spirulina (E3LIVE Blue Majik, Spirulina Extract for Healthy Joints and Inflammation Support, dietary supplement, 50 g fine powder), were prepared by weighing 50 mg of algae and adding 1 mL of methanol, followed by mixing using a MiniSpin centrifuge (3 min, 12 rpm) (Eppendorf, Hamburg, Germany). These extracts were analyzed by HPLC-ICP-MS to investigate the presence of arsenic species other than water-soluble forms. Additionally, both the fish and algae samples were spiked by adding 10 µL of each arsenolipid standard (AsFA 362 and AsHC360) to 1 mL of the prepared extract, resulting in an increase of 98 µg L^−1^ in the concentration of each arsenolipid.

## 3. Results and Discussion

### 3.1. Characterization of Synthesized Arsenolipids

#### 3.1.1. LC-MS Analysis

**Figure 2:**
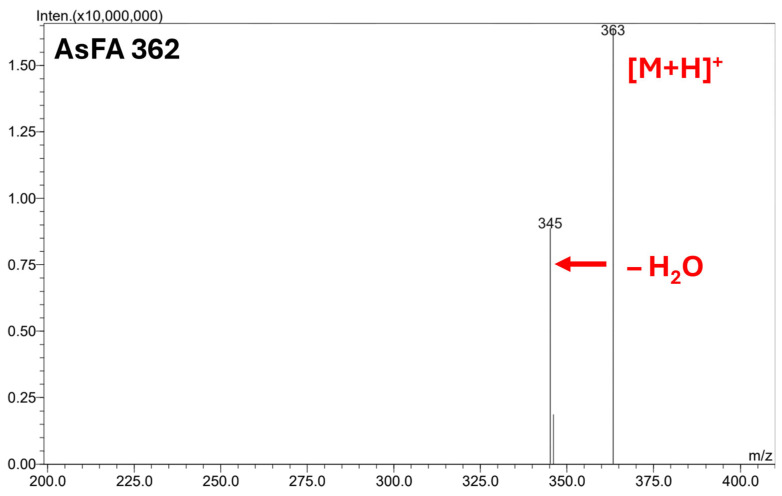

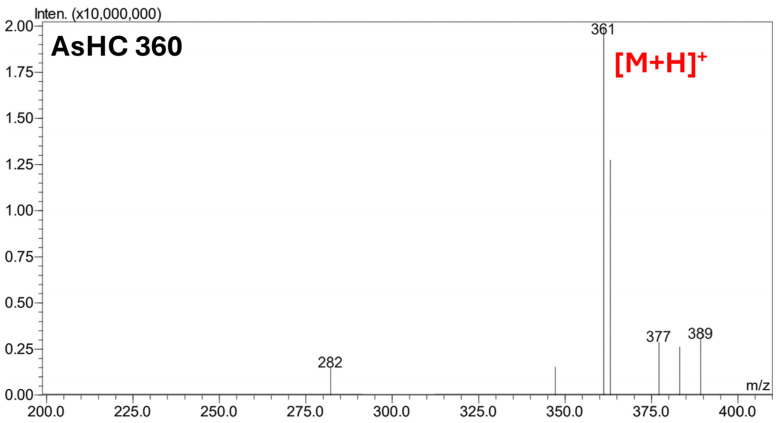
Mass spectra (LC-MS) of AsFA 362 and AsHC 360, which were prepared in methanol (1.5 mg mL^−1^).

The synthesized arsenolipids were dissolved in methanol and then analyzed by LC-MS. The obtained mass spectra confirmed the successful synthesis of the target arsenolipids (Figure 2). Protonated molecular ions ([M+H]^+^) were observed for both compounds: AsFA 362 showed the peak at *m*/*z* of 363, along with the fragment ion at *m*/*z* of 345 corresponding to the loss of water (−18 Da), while AsHC 360 showed the peak at *m*/*z* of 361.

#### 3.1.2. HPLC-ICP-MS Analysis

**Figure 3:**
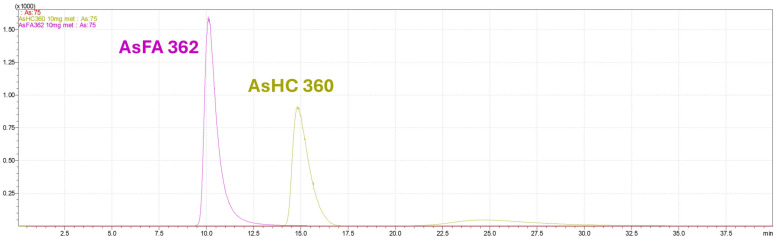
Overlaid chromatograms of AsFA 362 (10 mg L^−1^) and AsHC 360 (10 mg L^−1^), prepared in methanol.

The synthesized arsenolipids were dissolved in methanol and then analyzed by HPLC-ICP-MS (Table 1). The peaks were observed at different retention times: 10.098 min for AsFA 362 and 14.805 min for AsHC 360, as presented in Figure 3. The analyses performed using LC-MS and HPLC-ICP-MS confirmed the successful synthesis of both arsenolipids. The difference in their retention times indicates that the two compounds can be resolved under the applied chromatographic conditions and, therefore, can be analyzed simultaneously. Accordingly, they were used as the standards in the method development.

### 3.2. Method Development—Speciation of As(III), As(V), DMA, AsB, AsFA 362 and AsHC 360

The method development was conducted on a trial-and-error basis, with each issue and adjustment addressed immediately as it arose. This approach was taken to minimize gas and solvent consumption, given the long analysis times reported for arsenolipids (Table S1). Various analytical columns were evaluated, including the ion-exchange columns, primarily the anion-exchange columns, as well as the C8 and C18 reversed-phase columns (Table S6). Additionally, the retention times and peak areas of arsenic species obtained during the method development in the mixed standard solutions are presented in Table S7.

#### 3.2.1. Analyses with C18 Columns

Firstly, the short gradient was applied to observe how the water-soluble arsenic species, initially limited to As(III) and As(V), would be separated using the selected columns and the literature-reported conditions for arsenolipids [45,64,67,70] (Table S6—A1, B1, C1). The mobile phases consisted of 10 mmol L^−1^ ammonium acetate in water (A) and methanol (B) at pH = 6.0, with the flow rate of 1 mL min^−1^. Most of the effluent was diverted to waste prior to ICP-MS detection. On all three tested C18-based columns, As(III) and As(V) coeluted as a single peak. Under these conditions, arsenolipids (AsFA 362 and AsHC 360) were not detected and were only observed in the subsequent runs, indicating that a longer gradient was required. After extending the gradient to 15 min and switching to a different C18 column, the partial separation of As(III) and As(V) was achieved (Table S6—D1; Figure S1). However, arsenolipids were still not detected.

#### 3.2.2. Analyses with a C8 Column

The gradient was shortened to 11 min and the separation was performed on the C8-based column, while all the other parameters were kept unchanged (Table S6—E1). As(III) and As(V) were separated; however, arsenolipids were not detected, even in the subsequent analyses of the mobile phase (Figure S2). Increasing the injection volume to 500 µL did not improve detection. It was therefore hypothesized that arsenolipids were lost due to an excessive diversion of the effluent to waste. To address this, the mobile phase flow rate was reduced to 0.2 mL min^−1^. In addition, to mitigate the plasma instability caused by the high methanol content, the post-column dilution (0.6 mL min^−1^, 1% nitric acid) was introduced. Given the promising initial separation of As(III) and As(V), the C8 column was retained and further optimization was conducted using the extended gradients (Table S6—E2, E3; Figure S3). Under these conditions, As(III) and As(V) were not fully resolved but appeared as two partially separated peaks. However, the retention time of AsFA 362 was too close to the retention times of the water-soluble arsenic species, resulting in their incomplete separation. This effect became more pronounced upon the addition of AsB and DMA. The further optimization of the gradient elution conditions, as well as the application of the isocratic elution conditions, did not improve the overall separation (Table S6—E4–E6; Figure S4).

#### 3.2.3. Analyses of Samples

The method was applied to the selected samples to evaluate matrix effects on the extraction and the analysis, as well as its potential applicability (Table 1). Since the optimization described in Section 3.2.2 did not improve chromatographic performance, the gradient previously applied was used for the analysis of both the non-spiked and spiked samples (Table S6—E3; Figure S5). At this stage of the method development, AsFA 362 and AsHC 360 could not be unambiguously identified in the analyzed samples. However, the comparison of the chromatograms obtained for the non-spiked and spiked samples suggests their possible presence in the fish samples (Figure S6). In the analyzed salmon sample, both arsenolipids were tentatively observed. AsHC 360 was detected in the oil layer separated during the sample preparation and AsFA 362 was found in the methanol/water extract. In the tuna sample, arsenic species were not chromatographically resolved and only a single peak was observed. However, its peak area increased after the standard addition (Table 2). This observation is consistent with the coelution of arsenolipids with other arsenic species; however, the possibility that arsenolipids were absent from the sample cannot be excluded.

As the analyses were conducted qualitatively, accurate quantification of arsenolipid content in the fish samples was not possible. Nevertheless, assuming linearity between 0 mg L^–1^ and 1 mg L^−1^ (the concentration of the standards), the exploratory signal estimate suggested higher levels of AsFA 362 (~0.6 mg kg^–1^ dry mass) compared with AsHC 360 (~0.2 mg kg^–1^ dry mass) in the analyzed salmon samples. These values should be regarded as approximate estimates of arsenolipid content rather than definitive and accurate measurements. Taleshi et al. [54] reported arsenic content in the sashimi-grade tuna muscle tissue at the level of 5.9 mg kg^–1^ dry mass, with approximately 50% attributed to fat-soluble arsenic content. In another study of the tuna samples, Stiboller et al. [67] determined arsenolipid content in the brain (3.8–5.9 mg kg^−1^ dry mass, 34–51% of tAs) and the muscle (0.3–0.8 mg kg^−1^ dry mass, 9–14% of tAs). Compared with tuna, the proportion of fat-soluble arsenic relative to total arsenic (reported as 2.74 mg kg^−1^ dry mass) was lower in salmon, amounting to 6% [55].

#### 3.2.4. Analyses with More than One Column—Guard (Ion-Exchange) + C8

To improve the separation of the water-soluble arsenic species [As(III), As(V), AsB and DMA] and AsFA 362, a guard column was introduced prior to the analytical column. In addition, the post-column dilution flow rate was increased and the gradient program was adjusted (Table S6—F1). However, these modifications did not improve the separation—the water-soluble species coeluted as a single peak and were not fully resolved from AsFA 362 (Figure S7). Moreover, AsHC 360 was only observed in the subsequent run. The mobile phase A was then replaced with ammonium nitrate, based on our previously reported method for water-soluble arsenic speciation [80] (Table S6—F2). Despite the greater elution strength and the higher pH conditions, no improvement in separation was observed (Figure S8). Finally, extending the gradient to 40 min and increasing the time taken by the water-based mobile phase A at the beginning of the gradient also failed to separate AsFA 362 from the water-soluble arsenic species (Table S6—F3; Figure S9).

#### 3.2.5. Analyses with More than One Column—Guard (Ion-Exchange) + C18

Next, the guard column was retained, while the analytical column was changed from C8 to C18. The gradient was further extended (Table S6—G1). After it was observed that the mobile phase A (ammonium nitrate in water) was too strong, as As(III), As(V) and AsFA 362 coeluted as a single peak, the mobile phase A was switched back to ammonium acetate in water (Table S6—G2). This modification resulted in improved, although still incomplete, separation of AsFA 362 from As(III) and As(V). The water-soluble arsenic species continued to coelute as a single peak (Figure S10).

#### 3.2.6. Analyses with More than One Column—Guard (Ion-Exchange) + Anion-Exchange + C18

To improve the separation, the ion-exchange column was introduced between the guard column and the C18 column. The mobile phase flow rate was increased and the gradient program was adjusted (Table S6—H1). Although this configuration enabled the analysis under 40 min using the three-column setup, it did not improve the separation of AsFA 362 from the water-soluble arsenic species (Figure S11). The mobile phase A was then replaced with the stronger phase at a higher pH (Table S6—H2). This modification enabled the separation of AsFA 362 from As(III) and As(V). However, the latter were still obtained as a single peak (Figure S12). The stronger mobile phase reduced the retention of the water-soluble arsenic species while increasing the retention of arsenolipids, thereby improving their separation, but at the cost of longer analysis time (Figure S13). Extending the initial aqueous segment of the gradient did not improve the separation of the water-soluble arsenic species and mainly resulted in increased analysis time (Table S6—H3; Figure S14a). The mobile phase A was subsequently switched back to the weaker mobile phase, which improved the separation of the water-soluble arsenic species (Table S6—H4; Figure S14b). Next, the stronger mobile phase B was introduced to shorten the analysis time. However, this resulted in the less efficient separation of the water-soluble arsenic species after the column rinsing (Table S6—H5; Figure S14c).

#### 3.2.7. Analyses with More than One Column—C18 + Anion-Exchange + Guard (Ion-Exchange)

To investigate whether the separation of the water-soluble arsenic species could be further improved, the order of the columns was rearranged so that the C18 column was placed prior to the ion-exchange columns (Table S6—I1). This modification shortened the analysis time and decreased the retention times of both arsenolipids. However, it also increased the retention times of the water-soluble arsenic species and worsened their separation, resulting in the observation of only a single peak for these forms (Figures S15 and S16).

#### 3.2.8. Analyses with More than One Column—C18 + Anion-Exchange

Next, while maintaining the same analytical parameters, the IonPac columns were replaced with the Hamilton PRP-X110 column (Table S6—J1). This modification did not affect the analysis time, but it improved the chromatographic response, resulting in the higher and narrower peaks (Figures S17 and S18). To further optimize the analysis, the mobile phases containing different concentrations of ammonium acetate and adjusted to different pH values were evaluated (Table S6—J2, J3). It was observed that the use of both mobile phases containing 1 mmol L^−1^ ammonium acetate at pH = 6.0 negatively impacted the analysis, extending the analysis time to over 60 min (Figure S19a). When the mobile phase A remained unchanged and the mobile phase B was switched to 10 mmol L^−1^ ammonium acetate at pH = 6.0, the analysis time was reduced, although it still exceeded 60 min (Figure S19b). The best results were obtained when the pH of both mobile phases was increased from 6.0 to 9.2, while maintaining mobile phase A at 1 mmol L^−1^ ammonium acetate and increasing mobile phase B to 20 mmol L^−1^ ammonium acetate. Under these conditions, the analysis time was reduced to less than 60 min and the partial separation of As(III) and As(V) was achieved, demonstrating the potential of the method (Figure S19c). The brief summary and comparison of the arsenic speciation analysis methods described in the scientific literature (Tables S1 and S2) and the final developed method are presented in Table 3.

**Figure 4:**
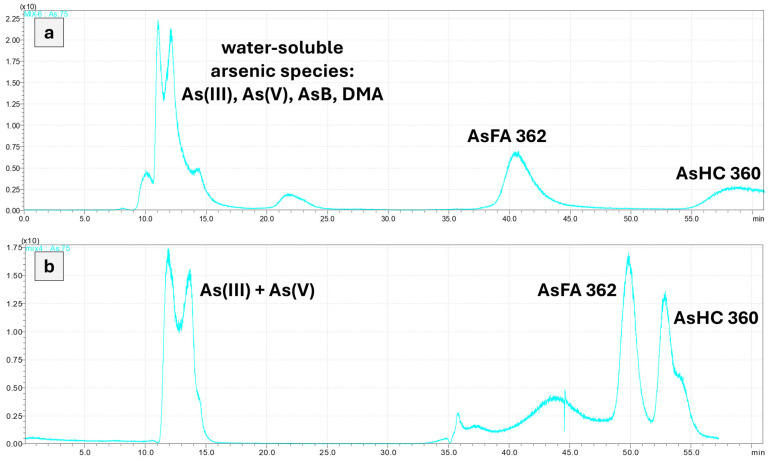
Chromatograms of (**a**) MIX of 6 species, mobile phases: A—10 mmol L^−1^ NH_4_OAc in water (pH = 6.0), B—10 mmol L^−1^ NH_4_OAc in methanol (pH = 6.0), columns: Thermo Scientific Dionex IonPac CG5A, Thermo Scientific Dionex IonPac AS22 and Shimadzu Shim-pack Scepter C18-120; (**b**) MIX of 4 species, mobile phases: A—1 mmol L^−1^ NH_4_OAc in water (pH = 9.2), B—20 mmol L^−1^ NH_4_OAc in methanol (pH = 9.2), columns: Shimadzu Shim-pack Scepter C18-120 and Hamilton PRP-X110.

Overall, the combination of the ion-exchange columns (Thermo Scientific Dionex IonPac CG5A with Dionex IonPac AS22, or just Hamilton PRP-X110) with the C18 column (Shimadzu Shim-pack Scepter C18-120) showed the greatest potential for the simultaneous analysis of the water-soluble arsenic species [As(III), As(V), DMA and AsB] and the fat-soluble arsenic species (arsenolipids: AsFA 362 and AsHC 360). Under the developed conditions, the arsenolipids were fully separated and eluted at longer retention times than the water-soluble arsenic species. However, we were not able to fully separate the water-soluble arsenic species, as presented in Figure 4. Although the method demonstrated potential, several limitations were identified. They included the long analysis time, the possibility of arsenolipids coelution [65,72] and their occurrence across a wide range of compounds in samples, as well as the lack of reported sample preparation methods suitable for the extraction of both water- and fat-soluble arsenic species. Therefore, further investment in the optimization was not justified within the scope of this study. Nevertheless, we believe that the performed analyses and the described step-by-step method development provide useful insights into arsenic speciation analysis. At present, HPLC-ICP-MS/ESI-MS remains the more reliable approach for the arsenolipids analysis, as it enables not only the determination of arsenolipids based on their retention time, but also their identification based on the mass spectrometric data.

## 4. Conclusions

The syntheses of the two arsenolipids, AsFA 362 and AsHC 360, were successfully completed, and the obtained products were analyzed by LC-MS and HPLC-ICP-MS. Subsequently, the two synthesized arsenolipids, alongside the water-soluble arsenic species, were used in a preliminary optimization study aimed at developing a method for the simultaneous arsenic speciation analysis. The arsenolipids were fully separated and eluted at longer retention times than the water-soluble arsenic species, demonstrating that the developed analytical method could be used to analyze them during the same measurement. However, the water-soluble arsenic species were only partially resolved. The combination of the ion-exchange columns with the C18 column showed the greatest potential, but the long analysis time, which could exceed 60 min with the introduction of even more arsenolipids, and no sample preparation methods reported for the extraction of both water- and fat-soluble arsenic species, did not justify the further investment in the method.

## Figures and Tables

**Table 1:** Operating parameters and details of the intermediate method applied for the analysis of AsFA 362 and AsHC 360 and for the analysis of fish and algae samples.

HPLC	
Analytical column	Shimadzu Shim-pack Scepter C8-120, 4.6 × 150 mm, 3 µm
Mobile phase	A: 10 mmol L^−1^ NH_4_OAc in water, pH = 6.0 B: 10 mmol L^−1^ NH_4_OAc in methanol, pH = 6.0
Gradient elution program	0–2 min, 0% B;
	2–4 min, 0 → 100% B;
	4–38 min, 100% B;
	38–39 min, 100 → 0% B;
	39–40 min, 0% B
Mobile phase flow rate	0.2 mL min^−1^
Post-column dilution of the effluent	1% HNO_3_
Post-column dilution flow rate	0.6 mL min^−1^
Injection volume	10 μL
Analysis time	40 min
**ICP-MS**	
Radio frequency power of the plasma	1.2 kW
Plasma gas (Ar) flow rate	15.0 L min^−1^
Nebulizer gas (Ar) flow rate	0.7 L min^−1^
Auxiliary gas (Ar) flow rate	1.2 L min^−1^
Nebulizer	Concentric (MicroMist)
Carrier gas (Ar) flow rate	0.85 L min^−1^
Sampling depth	7.0 mm
Spray chamber temperature	3 °C
Cell gas voltage	−21 V
Energy filter voltage	7.0 V
Cell gas (He) flow rate	6.0 mL min^−1^

**Table 2:** Retention times [min] and peak areas ± SD of AsFA 362 and AsHC 360 in spiked and non-spiked salmon and tuna samples.

Sample (*n = 3*)	Spiked Sample	Retention Time		Peak Area	
		AsFA 362	AsHC 360	AsFA 362	AsHC 360
Standard—1 mg L^−1^	—	10.276	15.385	2015.267 ± 34.412	891.038 ± 15.841
Salmon	No	9.198	—	47.701 ± 1.287	—
	Yes	9.813, 10.472	15.603	184.412 ± 4.425	47.365 ± 0.852
Salmon–oil	No	—	15.150	—	8.570 ± 0.171
	Yes	10.351	15.556	162.917 ± 3.747	69.980 ± 1.469
		**Arsenic species**		**Arsenic species**	
Tuna *	No	8.597		290.188 ± 8.125	
	Yes	9.227		462.396 ± 15.721	

* In the tuna sample, arsenic species were not chromatographically resolved and only a single peak was observed. Its retention time and peak area are presented in the table.

**Table 3:** Comparison of the final developed method with previously published arsenic speciation approaches.

	Analysis of Water-Soluble Arsenic Species	Analysis of Arsenolipids	Final Developed Method
Columns	Anion-exchange, anion and cation-exchange, C18	C8, C18, SuperPhenylHexyl	C18 + anion-exchange
Mobile phases	Salt or pyridine solutions, diluted acids or buffers	A: ammonium acetate or acetic/formic acid in water	A: 1 mmol L^−1^ ammonium acetate in water
		B: ammonium acetate or acetic/formic acid in alcohol	B: 20 mmol L^−1^ ammonium acetate in methanol
	pH = 2.3–9.5	pH = 6.0–9.2	pH = 9.2
Elution type	Gradient, isocratic	Gradient	Gradient
Analysis time	<35 min, under gradient elution conditions	<45 min	60 min
Determined arsenic species	Water-soluble arsenic species, such as As(III), As(V), AsB, AsC, DMA, MMA	Various arsenolipids, such as AsHC 360 and AsFA 362	Both water- and fat-soluble arsenic species: As(III), As(V), AsB, DMA, AsHC 360 and AsFA 362
Analytical technique	HPLC-ICP-MS	HPLC-ICP-MS/ESI-MS	HPLC-ICP-MS
Sources	[9,11,12,14,18,19,27,30,32,36, 41,69,70,78,79,80,81,82,83,84,85,86,87]	[45,48,49,50,51,64,67,68,69,70]	This study

## Data Availability

The original contributions presented in this study are included in the article/Supplementary Material. Further inquiries can be directed to the corresponding author.

## Supplementary Materials

The following supporting information can be downloaded at https://www.mdpi.com/article/10.3390/foods15132304/s1: Table S1: The details of the arsenolipids analyses reported in the literature, focusing on the methods used for the analysis of AsHC 360 and/or AsFA 362; Table S2: The details of the HPLC-based water-soluble arsenic species analyses recently reported in the literature; Table S3: Comparison of iododimethylarsine synthesis methods reported in the literature with the procedure developed in this work, which was adapted from the literature to accommodate the available laboratory equipment and capabilities; Table S4: Comparison of bis(dimethylarsenic) oxide synthesis methods reported in the literature with the procedure developed in this work, which was adapted from the literature to accommodate the available laboratory equipment and capabilities; Table S5: Comparison of arsenolipids synthesis methods reported in the literature with the procedure developed in this work, which was adapted from the literature to accommodate the available laboratory equipment and capabilities; Table S6: Columns, mobile phases and details of gradients used during method development; Table S7: Retention times [min] and peak areas of arsenic species in mixed standard solutions (1 mg L^−1^); Figure S1: Chromatogram of the mix of As(III) and As(V), 10 µg L^−1^, prepared in mobile phase A (10 mmol L^−1^ ammonium acetate in water, pH = 6.0); Figure S2: Chromatogram of the mix of As(III) and As(V), 10 µg L^−1^, prepared in the mobile phase A (10 mmol L^−1^ ammonium acetate in water, pH = 6.0); Figure S3: Chromatograms (a,b,e,f) of the synthesized AsFA 362 and AsHC 360, 1 mg L^−1^, prepared in methanol; chromatogram (c) of the mobile phase; chromatogram (d) of the mix of As(III) and As(V), 1 mg L^−1^, prepared in the mobile phase A (10 mmol L^−1^ ammonium acetate in water, pH = 6.0); chromatogram (g) of the mix of 4 arsenic species—As(III), As(V), AsFA 362 and AsHC 360, 1 mg L^−1^, prepared in methanol; chromatogram (h) of the mix of 6 arsenic species—As(III), As(V), AsB, DMA, AsFA 362 and AsHC 360, 1 mg L^−1^, prepared in methanol; and overlaid chromatograms (i,j); Figure S4: Chromatogram (a) of the sample—the marine algae sample with the addition of arsenolipids standards, extracted with methanol; chromatogram (b) of the mobile phase; chromatogram (c) of the mix of 4 arsenic species—As(III), As(V), AsFA 362 and AsHC 360, 1 mg L^−1^, prepared in methanol; chromatograms (d,i) of the mix of 6 arsenic species—As(III), As(V), AsB, DMA, AsFA 362 and AsHC 360, 1 mg L^−1^, prepared in methanol; chromatograms (e,f) of the synthesized AsFA 362 and AsHC 360, 1 mg L^−1^, prepared in methanol; and overlaid chromatograms (g,h); Figure S5: Chromatogram (a) of the mobile phase; chromatograms (b,c) of the marine algae sample without and with the addition of arsenolipids standards, extracted with methanol; chromatograms (e,g) of the red algae sample without and with the addition of arsenolipids standards, extracted with methanol; chromatograms (i,j) of the spirulina sample without and with the addition of arsenolipids standards, extracted with methanol; chromatograms (l,m) of the salmon sample without and with the addition of arsenolipids standards, extracted with methanol/water; chromatograms (o,p) of the salmon–oil, which separated during the salmon sample preparation, without and with the addition of arsenolipids standards; chromatograms (s,t) of the tuna sample without and with the addition of arsenolipids standards, extracted with methanol/water; and overlaid chromatograms (d,f,h,k,n,r,u); Figure S6: Comparison of the chromatograms obtained for the fish samples without and with the addition of the arsenolipids standards—AsFA 362 and AsHC 360; Figure S7: Chromatogram (a) of the mix of 6 arsenic species—As(III), As(V), AsB, DMA, AsFA 362 and AsHC 360, 1 mg L^−1^, prepared in methanol; chromatogram (b) of the mix of 4 arsenic species—As(III), As(V), AsFA 362 and AsHC 360, 1 mg L^−1^, prepared in methanol; chromatogram (c) of the mobile phase; and chromatogram (d) of the mix of As(III) and As(V), 1 mg L^−1^, prepared in the mobile phase A (10 mmol L^−1^ ammonium acetate in water, pH = 6.0); Figure S8: Chromatogram (a) of the mix of 6 arsenic species—As(III), As(V), AsB, DMA, AsFA 362 and AsHC 360, 1 mg L^−1^, prepared in methanol; chromatogram (b) of the mobile phase; chromatogram (c) of the synthesized AsFA 362, 1 mg L^−1^, prepared in methanol; and chromatogram (d) of the mix of As(III) and As(V), 1 mg L^−1^, prepared in the mobile phase A (10 mmol L^−1^ ammonium acetate in water, pH = 6.0); Figure S9: Chromatogram (a) of the mix of 6 arsenic species—As(III), As(V), AsB, DMA, AsFA 362 and AsHC 360, 1 mg L^−1^, prepared in methanol; chromatogram (b) of the mix of 4 arsenic species—As(III), As(V), AsFA 362 and AsHC 360, 1 mg L^−1^, prepared in methanol; chromatogram (c) of the mix of the synthesized AsFA 362 and AsHC 360, 1 mg L^−1^, prepared in methanol; and overlaid chromatograms (d,e); Figure S10: Chromatogram of the mix of 4 arsenic species—As(III), As(V), AsFA 362 and AsHC 360, 1 mg L^−1^, prepared in methanol; Figure S11: Chromatogram of the mix of 4 arsenic species—As(III), As(V), AsFA 362 and AsHC 360, 1 mg L^−1^, prepared in methanol; Figure S12: Chromatogram of the mix of 4 arsenic species—As(III), As(V), AsFA 362 and AsHC 360, 1 mg L^−1^, prepared in methanol; Figure S13: Overlaid chromatograms, which were presented in Figures S11 and S12; Figure S14: Chromatograms (a–c) of the mix of 6 arsenic species—As(III), As(V), AsB, DMA, AsFA 362 and AsHC 360, 1 mg L^−1^, prepared in methanol; Figure S15: Chromatogram of the mix of 4 arsenic species—As(III), As(V), AsFA 362 and AsHC 360, 1 mg L^−1^, prepared in methanol; Figure S16: Overlaid chromatograms, which were presented in Figures S14b and S15; Figure S17: Chromatogram of the mix of 4 arsenic species—As(III), As(V), AsFA 362 and AsHC 360, 1 mg L^−1^, prepared in methanol; Figure S18: Overlaid chromatograms, which were presented in Figures S15 and S17; Figure S19: Chromatograms (a–c) of the mix of 4 arsenic species—As(III), As(V), AsFA 362 and AsHC 360, 1 mg L^−1^, prepared in methanol. Refs. [9,11,12,14,18,19,27,30,32,36,41,45,48,49,50,51,64,67,68,69,70,73,74,78,79,80,81,82,83,84,85,86,87] are cited in the Supplementary Materials.
