# Supplementary material for: Simultaneous Determination of Water- and Fat-Soluble Arsenic Species by HPLC-ICP-MS in Food Samples: A Pilot Study
Source: Foods. 2026 Jun 28;15(13):2304. doi: 10.3390/foods15132304 (PMC13361988; doi:10.3390/foods15132304)
Supplement: Supplementary file 1 [file foods-15-02304-s001.zip › foods-4378617-supplementary.pdf]

Table of contents

Table S1. The details of the arsenolipids analyses reported in literature, focusing on the methods used for the analysis of AsHC 360 and/or AsFA 362. 3

Table S2. The details of the HPLC-based water-soluble arsenic species analyses recently reported in literature. 5

Table S3. Comparison of iododimethylarsine synthesis methods reported in the literature with the procedure developed in this work, which was adapted from the literature to accommodate the available laboratory equipment and capabilities. 9

Table S4. Comparison of bis(dimethylarsenic) oxide synthesis methods reported in the literature with the procedure developed in this work, which was adapted from the literature to accommodate the available laboratory equipment and capabilities. 10

Table S5. Comparison of arsenolipids synthesis methods reported in the literature with the procedure developed in this work, which was adapted from the literature to accommodate the available laboratory equipment and capabilities. 11

Table S6. Columns, mobile phases and details of gradients used during method development. 12

Table S7. Retention times [min] and peak areas of arsenic species in mixed standard solutions (1 mg L^{-1}). 15

Arsenolipids synthesis 16

Iododimethylarsine synthesis 16

Bis(dimethylarsenic) oxide synthesis 16

15-(Dimethylarsinyl)pentadecanoic, AsFA 362, synthesis 16

Additional observations 17

Method development 19

Figure S1. Chromatogram of the mix of As(III) and As(V), 10 µg L^{-1}, prepared in mobile phase A (10 mmol L^{-1} ammonium acetate in water, pH=6.0). 19

Figure S2. Chromatogram of the mix of As(III) and As(V), 10 µg L^{-1}, prepared in the mobile phase A (10 mmol L^{-1} ammonium acetate in water, pH=6.0). 19

Figure S3. Chromatograms (a, b, e, f) of the synthesized AsFA 362 and AsHC 360, 1 mg L^{-1}, prepared in methanol; chromatogram (c) of the mobile phase; chromatogram (d) of the mix of As(III) and As(V), 1 mg L^{-1}, prepared in the mobile phase A (10 mmol L^{-1} ammonium acetate in water, pH=6.0); chromatogram (g) of the mix of 4 arsenic species — As(III), As(V), AsFA 362 and AsHC 360, 1 mg L^{-1}, prepared in methanol; chromatogram (h) of the mix of 6 arsenic species — As(III), As(V), AsB, DMA, AsFA 362 and AsHC 360, 1 mg L^{-1}, prepared in methanol; and overlaid chromatograms (i, j). 20

Figure S4. Chromatogram (a) of the sample — the marine algae sample with the addition of arsenolipids standards, extracted with methanol; chromatogram (b) of the mobile phase; chromatogram (c) of the mix of 4 arsenic species — As(III), As(V), AsFA 362 and AsHC 360, 1 mg L^{-1}, prepared in methanol; chromatograms (d, i) of the mix of 6 arsenic species — As(III), As(V), AsB, DMA, AsFA 362 and AsHC 360, 1 mg L^{-1}, prepared in methanol; chromatograms (e, f) of the synthesized AsFA 362 and AsHC 360, 1 mg L^{-1}, prepared in methanol; and overlaid chromatograms (g, h). 21

Figure S5. Chromatogram (a) of the mobile phase; chromatograms (b, c) of the marine algae sample without and with the addition of arsenolipids standards, extracted with methanol; chromatograms (e, g) of the red algae sample without and with the addition of arsenolipids standards, extracted with methanol; chromatograms (i, j) of the spirulina sample without and with the addition of arsenolipids standards, extracted with methanol; chromatograms (l, m) of the salmon sample without and with the addition of arsenolipids standards, extracted with methanol/water; chromatograms (o, p) of the salmon–oil, which separated during

the salmon sample preparation, without and with the addition of arsenolipids standards; chromatograms (s, t) of the tuna sample without and with the addition of arsenolipids standards, extracted with methanol/water; and overlaid chromatograms (d, f, h, k, n, r, u). 23

Figure S6. Comparison of the chromatograms obtained for the fish samples without and with the addition of the arsenolipids standards – AsFA 362 and AsHC 360. 24

Figure S7. Chromatogram (a) of the mix of 6 arsenic species — As(III), As(V), AsB, DMA, AsFA 362 and AsHC 360, 1 mg L^{-1}, prepared in methanol; chromatogram (b) of the mix of 4 arsenic species — As(III), As(V), AsFA 362 and AsHC 360, 1 mg L^{-1}, prepared in methanol; chromatogram (c) of the mobile phase; and chromatogram (d) of the mix of As(III) and As(V), 1 mg L^{-1}, prepared in the mobile phase A (10 mmol L^{-1} ammonium acetate in water, pH=6.0). 25

Figure S8. Chromatogram (a) of the mix of 6 arsenic species — As(III), As(V), AsB, DMA, AsFA 362 and AsHC 360, 1 mg L^{-1}, prepared in methanol; chromatogram (b) of the mobile phase; chromatogram (c) of the synthesized AsFA 362, 1 mg L^{-1}, prepared in methanol; and chromatogram (d) of the mix of As(III) and As(V), 1 mg L^{-1}, prepared in the mobile phase A (10 mmol L^{-1} ammonium acetate in water, pH=6.0). 25

Figure S9. Chromatogram (a) of the mix of 6 arsenic species — As(III), As(V), AsB, DMA, AsFA 362 and AsHC 360, 1 mg L^{-1}, prepared in methanol; chromatogram (b) of the mix of 4 arsenic species — As(III), As(V), AsFA 362 and AsHC 360, 1 mg L^{-1}, prepared in methanol; chromatogram (c) of the mix of the synthesized AsFA 362 and AsHC 360, 1 mg L^{-1}, prepared in methanol; and overlaid chromatograms (d, e). 26

Figure S10. Chromatogram of the mix of 4 arsenic species — As(III), As(V), AsFA 362 and AsHC 360, 1 mg L^{-1}, prepared in methanol. 26

Figure S11. Chromatogram of the mix of 4 arsenic species — As(III), As(V), AsFA 362 and AsHC 360, 1 mg L^{-1}, prepared in methanol. 27

Figure S12. Chromatogram of the mix of 4 arsenic species — As(III), As(V), AsFA 362 and AsHC 360, 1 mg L^{-1}, prepared in methanol. 27

Figure S13. Overlaid chromatograms, which were presented in figures S11 and S12. 27

Figure S14. Chromatograms (a–c) of the mix of 6 arsenic species — As(III), As(V), AsB, DMA, AsFA 362 and AsHC 360, 1 mg L^{-1}, prepared in methanol. 28

Figure S15. Chromatogram of the mix of 4 arsenic species — As(III), As(V), AsFA 362 and AsHC 360, 1 mg L^{-1}, prepared in methanol. 29

Figure S16. Overlaid chromatograms, which were presented in figures S14.b and S15. 29

Figure S17. Chromatogram of the mix of 4 arsenic species — As(III), As(V), AsFA 362 and AsHC 360, 1 mg L^{-1}, prepared in methanol. 29

Figure S18. Overlaid chromatograms, which were presented in figures S15 and S17. 30

Figure S19. Chromatograms (a–c) of the mix of 4 arsenic species — As(III), As(V), AsFA 362 and AsHC 360, 1 mg L^{-1}, prepared in methanol. 31

References 32

Table S1. The details of the arsenolipids analyses reported in literature, focusing on the methods used for the analysis of AsHC 360 and/or AsFA 362.

Column	Mobile Phase	Flow rate [mL min^{-1}]	Elution type	Determined arsenolipids	Analytical technique	Source
ACE UltraCore SuperC18 4.6×250 mm, 5 µm	A: 20 mmol L^{-1} ammonium acetate in water, pH=9.2<br>B: 20 mmol L^{-1} ammonium acetate in methanol, pH=9.2	1.0	Gradient<br>0–15 min, 20→100% B; 15–35 min, 100% B; 35–35.1 min, 100→50% B; 35.1–40 min, 50% B	AsHC 332, AsHC 358, **AsHC 360**, AsHC 388, **AsFA 362**, AsFA 390, AsSugPL 720, AsSugPL 958, AsSugPL 986, AsSugPL 1014, AsSugPL 1042, AsSugPL 1070	RP-HPLC-ICP-MS/ ESI-MS-MS	[45]
ACE UltraCore 5 SuperPhenylHexyl 4.6×250 mm, 5 µm	A: 25 mmol L^{-1} ammonium acetate in water, pH=9.2<br>B: 25 mmol L^{-1} ammonium acetate in methanol, pH=9.2	0.9	Gradient<br>0–1 min, 0→20% B; 1–2 min, 20→60% B; 2–12 min, 60→100% B; 12–25 min, 100% B; 25.1–30 min, 0% B	AsHC 332, **AsHC 360**	HPLC-ICPMS/ ESMS	[70]
ACE UltraCore 5 SuperPhenylHexyl 4.6×250 mm, 5 µm	A: 25 mmol L^{-1} ammonium acetate in water, pH=9.2<br>B: 25 mmol L^{-1} ammonium acetate in methanol, pH=9.2	1.0	Gradient<br>0–2 min, 0→20% B; 2–4 min, 20→80% B; 4–12 min, 80→100% B; 12–17 min, hold; 17.1–25 min, 100→0% B	**AsFA 362**, AsFA 388, AsFA 418, AsHC 332, **AsHC 360**, AsHC 444	HPLC-ICP-MS	[67]
ACE SuperHexylPhenyl 4.6×250 mm, 5 µm	A: 25 mmol L^{-1} acetic acid in water, pH=9.2<br>B: 25 mmol L^{-1} acetic acid and 0.5% NH_3 in methanol	1.0	Gradient<br>0–25 min, 40→100% B; 25–35 min, 100% B; 35–35.5 min, 100→40% B; 35.5–42 min, 40% B	AsHC 332, **AsHC 360**, AsIsop 408, AsIsop 422, AsIsop 546, AsPL 718, AsPL 720, AsPL 958, AsPL 978, AsPL 980, AsPL 982, AsPL 984, AsPL 986, AsPL 1014	HPLC-ICPMS/ ESMS	[49]
Shodex<br>Asahipak C8 4.6×150 mm, 5 µm	A: 0.1% formic acid in water<br>B: 0.1% formic acid in methanol	0.5	Gradient<br>0–15 min, 50→100% B; 15–23 min, 100% B; 23–23.1 min, 100→50% B; 23.1–30 min, 50% B	AsHC 332, **AsFA 362**, AsFE 376, AsFE 390, AsFE 404, AsFE 418, Thioxo-FA362, Aslipid-586, Aslipid-612, Aslipid-732, Aslipid-746, Aslipid-760	HPLC-ICPMS/ ESMS<br>HPLC-HR-ESMS	[69]

Shodex Asahipak ODP-50 4.6×150 mm; 5 µm	A: 0.1% formic acid in water<br>B: 0.1% formic acid in methanol	0.5	Gradient<br>0−25 min, 60→100% B;<br>25−32 min, 100% B;<br>32−32.1 min, 100→60% B;<br>32.1−40 min, 60% B	AsHC 332, **<u>AsHC 360</u>**, AsSugPhytol, AsPL 958, AsPL 978, AsPL 1006	HPLC-ICPMS/ ES-QQQ-MS	[50]
Shodex Asahipak ODP-50 4D 4.6×150 mm ODP-50G 4A 4.6×10 mm), 5 µm	A: 0.1% formic acid in water<br>B: 0.1% formic acid in ethanol	0.5	Gradient<br>0–1 min, 30% B;<br>1–33 min, 30→100% B;<br>33–35 min, 100% B;<br>35–45 min, 30% B	AsFA 334, **<u>AsFA 362</u>**, AsFA 388, AsFA 390, AsFA 436, AsFA 448, AsFA 528, AsHC 330, AsHC 332, AsHC 346, AsHC 358, **<u>AsHC 360</u>**, AsHC 404, AsPC 885, AsPC 911, AsPC 939, AsPC 985, AsPC 997	HPLC-ICP-MS/ ESIMS	[68]
Agilent Zorbax SB-C8 1×50 mm, 3.5 µm	A: 10 mmol L^{-1} ammonium acetate in water, pH=6.0<br>B: ethanol	0.2	Gradient<br>0−25 min, 35→95% B	**<u>AsFA 362</u>**, AsFA 388, AsFA 418, AsHC 332, **<u>AsHC 360</u>**, AsHC 444	HPLC-ICP-MS/ ESI-MS	[64]
Agilent Zorbax Eclipse XDB-C8 4.6×150 mm, 5 µm	A: 0.1% formic acid in water<br>B: 0.1% formic acid in ethanol	1.0	Gradient<br>0−3 min, 70% B;<br>3−10 min, 90% B;<br>10−20 min, 90% B	AsHC 332, **<u>AsHC 360</u>**, AsPL 958, AsPL 986, AsPL 1014, AsPL 1042, AsPL 1070	RP-HPLC-ICPMS/ HR-ESMS	[48]
Agilent Eclipse XDB-C18 4.6×150 mm, 5 µm	A: 0.1% formic acid in water<br>B: 0.1% formic acid in methanol	1.0	Gradient<br>20 min, 70→100% B;<br>20 min, hold<br>———<br>Gradient<br>0−20 min, 0→100% B;<br>25 min, hold	AsHC 304, AsHC 332, AsHC 346, AsHC 358, **<u>AsHC 360</u>**, AsHC 402, AsHC 388, AsPL 954, AsPL 980, AsPL 930, AsPL 944, AsPL 956, AsPL 982, AsPL 958, AsPL 972, AsPL 984, AsPL 986, AsPL 1012, AsPL 1000, AsPL 1014, AsPL 1028	HPLC-ICP-MS<br>———<br>HPLC-ICP-MS/ ESI-MS	[51]

Table S2. The details of the HPLC-based water-soluble arsenic species analyses recently reported in literature.

Column	Mobile Phase	Flow rate [mL min^{-1}]	Elution type	Determined arsenic species	Analytical technique	Source
Thermo Scientific<br>Dionex IonPac AS7 4×250 mm	A: 5 mmol L^{-1} ammonium carbonate, pH=9.5<br>B: 100 mmol L^{-1} ammonium carbonate, pH=9.5	1.0	-	As(III), As(V), AsB, AsC, DMA, MMA	HPLC-ICP-MS	[32]
Thermo Scientific<br>Dionex IonPac AS7 4×250 mm	A: 0.36 g L^{-1} ammonium carbonate, pH=9.12<br>B: 8.00 g L^{-1} ammonium carbonate, pH=9.18	1.0	Gradient<br>0–3 min, 0% B;<br>3–3.5 min, 0→80% B;<br>3.5–6.5 min, 80→100% B;<br>6.5–7.8 min, 100→0% B;<br>7.8–15 min, hold	As(III), As(V), AsB, AsC, DMA, MMA	HPLC-ICP-MS	[30]
Thermo Scientific<br>IonPac AS7 2×150 mm<br>IonPac AG7 2×50 mm	A: 0.5 mmol L^{-1} ammonium carbonate, 3% MeOH, pH=9.3<br>B: 50 mmol L^{-1} ammonium carbonate, 3% MeOH, pH=9.3	0.5	Gradient<br>0–2 min, 0% B;<br>2.5–8 min, 100% B;<br>8.5–10 min, 0% B	As(III), As(V), DMA, MMA	HPLC-ICP-MS	[78]
Thermo Scientific<br>Dionex IonPac AS7 4×250 mm	A: 0.5 mmol L^{-1} ammonium carbonate<br>B: 50 mmol L^{-1} ammonium carbonate, 100 mmol L^{-1} ammonium nitrate, 4% methanol	1.0	Gradient<br>0 min, 0% B;<br>5 min, 0% B;<br>15 min, 100% B;<br>25 min, 100% B;<br>30 min, 0% B;<br>35 min, 0% B	As(III), As(V), AsB, AsC, DMA, MMA	HPLC-ICP-MS	[87]
Thermo Scientific<br>Dionex IonPac AS19	ammonium carbonate	-	-	As(III), As(V), AsB, AsC, DMA, MMA	HPLC-ICP-MS	[84]
Thermo Scientific<br>Dionex IonPac AS22<br>Dionex IonPac CG5A	A: 1 mmol L^{-1} ammonium nitrate, 1% methanol, pH=9<br>B: 75 mmol L^{-1} ammonium nitrate, 1% methanol, pH=9	1.0	Gradient<br>0–2 min, 0% B;<br>2–4 min, 0→100% B;<br>4–10 min, 100% B;<br>10–12 min, 0% B	As(III), As(V), AsB, DMA, MMA	HPLC-ICP-MS	[80]

Hamilton<br>PRP-X100 PEEK<br>4.6×250 mm, 10 µm<br>3×8 mm, 10 µm	50 mmol L^{-1} ammonium carbonate,<br>0.2 mmol L^{-1} EDTA, 1% methanol, pH=8.5	0.8	Isocratic	As(III), As(V), AsB, AsC, DMA, MMA	HPLC-ICP-MS	[27]
Hamilton<br>PRP-X100 PEEK<br>2.1×250 mm, 5 µm	A: 50 mmol L^{-1} ammonium bicarbonate, 3% methanol<br>B: ultrapure water<br>pH= 10.3	0.35	Isocratic (85:15, A:B)	iAs, AsB, DMA, MMA	HPLC-ICP-MS	[19]
Hamilton<br>PRP-X100<br>4.1×150 mm, 5 µm<br>PRP-X100<br>4.6×20 mm, 12–20 µm	35 mmol L^{-1} ammonium bicarbonate, 5% methanol, pH=8.25	0.8 (0.00–4.00 min);<br>1.5 (4.01–19.00 min)	Isocratic	As(III), As(V), AsB, DMA, MMA	HPLC-ICP-MS	[81, 82]
Hamilton<br>PRP-X100<br>4.6×150 mm, 5 µm	20 mmol L^{-1} ammonium phosphate, pH=6.0	1.0	Isocratic	iAs, DMA, MMA	HPLC-ICP-MS	[86]
Hamilton<br>PRP-X100	10 mmol L^{-1} diammonium phosphate and 10 mmol L^{-1} ammonium nitrate, pH=6.2	-	Isocratic	As(III), As(V), DMA, MMA	HPLC-ICP-MS	[11]
Hamilton<br>PRP-X100	20 mmol L^{-1} monopotassium phosphate, pH=6.1	1.0	Isocratic	As(III), As(V), DMA, MMA	HPLC-HG-AFS	[12]
Hamilton<br>PRP-X100<br>4×150 mm, 10 µm	3.5 mmol L^{-1} malonic acid with 1% H_2O_2, pH=5.6	1.0	-	iAs, DMA, MMA	HPLC-ICP-MS/MS	[18]
Hamilton<br>PRP-X200<br>4×250 mm, 10 µm	10 mmol L^{-1} pyridine, pH=2.6			AsB		

Hamilton PRP-X100	20 mmol L^{-1} phosphate buffer, pH=6.0	-	-	iAs, AsB, AsC, DMA, MMA, TMAO, TETRA, $C_5H_{13}AsNO^+$	HPLC-ICP-MS	[85]
Dr. Maisch Reprosil-XR 300 SCX	10 mmol L^{-1} pyridine solution, pH=2.3					
	ammonium formate buffer			$C_5H_{13}AsNO^+$ (arsenobetaine amide)	HPLC-ICP-MS/ HR ESI-MS	
Hamilton PRP-X100 4.6×150 mm, 5 µm	5 mmol L^{-1} malonic acid, pH=5.6	1.0	Isocratic	As(V), DMA, MMA	HPLC-ICPMS	[70]
Agilent Zorbax 300SCX 4.6×250, 5 µm	20 mmol L^{-1} ammonium formate, pH=3.5	1.0	Isocratic	AsB, AsC, DMA, TMAO, TETRA		
Hamilton PRP-X100 4.1×250 mm, 10 µm PRP-X100	A: 10 mmol L^{-1} ammonium acetate, 1% MeOH, pH=8.3 B: 40 mmol L^{-1} ammonium oxalate, 1% MeOH, pH=8.3	1.0	Gradient 0.00 min, 0% B; 2.00 min, 0% B; 5.00 min, 100% B; 5.30 min, 0% B; 13.00 min, 0% B	iAs, DMA, MMA	HPLC-ICP-MS	[79]
Hamilton PRP-X100	A: 15 mmol L^{-1} ammonium carbonate B: 50 mmol L^{-1} ammonium carbonate	1.0	Gradient 0–6 min, 0% B 6–30 min, 100% B	As(III), As(V), AsB, AsC, DMA, MMA	HPLC-ICP-MS	[14]
Hamilton PRP-X100 4.6×150 mm, 5 µm	A: 200 mmol L^{-1} ammonium carbonate, 3% methanol B: 0.5 mmol L^{-1} ammonium nitrate, 3% methanol	1.0	Gradient 0–2 min, 100% B; 2–3 min, 100→50% B; 3–8 min, 50% B; 8–9 min, 50→0% B; 9–12 min, 0% B; 12–13 min, 0→100% B; 13–15 min, 100% B	As(III), As(V), AsB, AsC, DMA, MMA	HPLC-ICP-MS	[9]

Hamilton<br>PRP-X100<br>4.1×250 mm, 10 µm<br>PRP-X100<br>2.3×25 mm, 12–20 µm	A: 20 mmol L^{-1} ammonium carbonate, pH=9.0<br>B: deionized water<br>C: 20 mmol L^{-1} ammonium carbonate, pH=10.3	1.0	Gradient<br>0–7 min, 5% A and 95% B;<br>7–14 min, 100% B;<br>14–20 min, 100% C;<br>20–30 min, 5% A and 95% B	As(III), As(V), DMA, MMA	HPLC-UV-Thermo-oxidation-AFS	[36]
Hamilton<br>PRP-X100<br>4.1×250 mm, 10 µm	A: 20 mmol L^{-1} ammonium bicarbonate<br>B: 50 mmol L^{-1} ammonium carbonate	1.0	Gradient<br>0.00–4.00 min, 0% B;<br>4.00–4.01 min, 0→100% B;<br>4.01 – 14.00 min, 100% B	As(III), As(V), AsB, AsC, DMA, MMA	HPLC-ICP-MS/MS	[41]
Hamilton<br>PRP-X100	A: 20 mmol L^{-1} malonic acid, pH=5.6<br>B: water	1.0	Gradient<br>0–9 min, 95→0% B;<br>9–11 min, 0% B;<br>11–11.1 min, 0→95% B;<br>11.1–15 min, 95% B	As(III), As(V), DMA, MMA	HPLC-ICP-MS	[69]
Ionosphere C5<br>3×200 mm, 5 µm	A: 10 mmol L^{-1} pyridine, pH=2.8	1.0	Isocratic			
Osaka Soda<br>Capcell Pak C18 MG<br>4.6×150 mm, 5 µm<br>Capcell Pak<br>3.0×4 mm, 5 µm	2 mmol L^{-1} 1-octanesulfonic acid sodium salt, 2 mmol L^{-1} malonic acid, 4 mmol L^{-1} tetramethylammonium hydroxide solution, pH= 4.13	1.0	Isocratic	As(III), As(V), DMA, MMA	HPLC-ICP-MS	[83]

Table S3. Comparison of iododimethylarsine synthesis methods reported in the literature with the procedure developed in this work, which was adapted from the literature to accommodate the available laboratory equipment and capabilities.

	**Arroyo-Abad et al., 2016 [74]**	**Taleshi et al., 2014 [64]**	**Our study**
**Heating and stirring**	4 hours, reduced from 24 hours		22 hours
**Temperature**	Room temperature		Room temperature
**Reagents**	DMA (2 g) in water (8 mL),<br>KI (6.6 g),<br>$NaHSO_3$ (1 g),<br>conc. HCl (10 mL)		DMA (2 g), KI (6.6 g) and $NaHSO_3$ (1 g) in water (8 mL),<br>conc. HCl (10 mL)
**Observations**	A dark brown layer was observed at the bottom of the reaction mixture		Color of the reaction mixture changed from yellow to brownish orange.<br>Three layers were observed in the flask — a dark bottom layer, covered by a light layer, and an orange solution
**Extraction**	Only the dark bottom layer		Whole reaction mixture
	Chloroform (40 mL) and water (60 mL)		Chloroform (20 mL) and water (60 mL), once, and chloroform (40 mL) and water (60 mL), twice
	-		Vigorous shaking
**Treatment of chloroform layer**	Washed and dried over Na_2SO_4		Combined and dried over Na_2SO_4
	Concentrated using a TurboVap® II concentrator		Evaporated on a rotary evaporator
**Product**	Clear yellow oil		Clear brownish yellow oil
	-		Distilled — clear yellow oil (72 °C, 90 mbar)

Table S4. Comparison of bis(dimethylarsenic) oxide synthesis methods reported in the literature with the procedure developed in this work, which was adapted from the literature to accommodate the available laboratory equipment and capabilities.

	**Arroyo-Abad et al., 2016 [74]**	**Taleshi et al., 2014 [64]**	**Our study**
**Argon**	Under argon	Under argon	Under argon
**Reagents**	Iododimethylarsine (0.5 mL, freshly prepared),<br>NaOH (5 mL, 10 M, 2 °C)	Iododimethylarsine (3.0 mmol),<br>NaOH (0.3 mL, 10 M, 3.0 mmol, 2 °C)	Iododimethylarsine (0.5 mL, distilled),<br>NaOH (5 mL, 10 M, 2 °C)
**Stirring time**	-	10 minutes	15 minutes
**Extra treatment**	-	Top layer was separated from the aqueous layer and covered with 10 M NaOH (0.3 mL, 3.0 mmol)	-
**Observations**	The formation of BDMAO was observed at the surface of the solution as oily layer	-	Clear oil drops formed on the surface, during mixing emulsion was formed

Table S5. Comparison of arsenolipids synthesis methods reported in the literature with the procedure developed in this work, which was adapted from the literature to accommodate the available laboratory equipment and capabilities.

	**Arroyo-Abad et al., 2016 [74]**	**Taleshi et al., 2014 [64]**	**Our study**
**Synthesis of**	AsHC 374 and *AsFA 264*	AsHC 360, AsFA 362, and others	AsHC 360, AsFA 362
**Reagents**	A suspension of 1-bromooctadecane (1.7 g, 5 mmol) in ethanol (10 mL)	A suspension of a bromo compound (0.5 mmol) in ethanol (0.5 mL)	A freshly prepared suspension of 15-bromopentadecanoic acid (1.6 g, 5 mmol) or 1-bromoheptadecane (1.6 g, 5 mmol) in ethanol (10 mL)
**Stirring and heating time**	Stirred and refluxed (78 °C) for 12 hours	Stirred and heated (80 °C) overnight	Stirred and heated overnight, in an oil bath (80 °C)
**Extraction**	Diluted with water and washed with diethyl ether	Diluted with water and washed with ether	Diluted with water (to the total volume of 40 mL) and washed with diethyl ether (40 mL), thrice
**pH**	Neutral pH with 6M HCl *or pH=1 and saponification procedure*	pH=3.5 (AsFA) or neutral pH (AsHC), with 6 M HCl	pH=3.5 (AsFA) or pH=7 (AsHC), with 6M HCl
**Extraction**	The aqueous layer was extracted with chloroform, then the organic layer was washed	The aqueous layer was extracted with chloroform, then the organic layer was washed with water	Extracted thrice with chloroform (40 mL), chloroform layers were combined and washed thrice with deionized water (120 mL). The chloroform layer was dried over Na_2SO_4
**Treatment of chloroform layer**	Evaporated in vacuum	Evaporated in vacuum	Evaporated on a rotary evaporator
**Treatment of residue**	Cleaned by silica gel chromatography (home-made column) - ethyl acetate/methanol	Crystallized from ethyl acetate	Crystallized from ethyl acetate, then filtered under pressure
**Product**	White solid	White solid	White solid
**Yield**	58% (AsHC 374), *and 50% (AsFA 264)*	63% (AsHC 360), 88% (AsFA 362)	44.1 % (AsFA 362), 22.2 % (AsHC 360)

Table S6. Columns, mobile phases and details of gradients used during method development.

Columns		Mobile phases and gradient details — time, percentage of mobile phase B
Supelco SUPELCOSIL LC-18<br>4.6×150 mm, 5 µm	A1	A — 10 mmol L^{-1} ammonium acetate in water (pH=6.0), B — 10 mmol L^{-1} ammonium acetate in methanol (pH=6.0)<br>Gradient (1 mL min^{-1})<br>0–2 min, 0% B; 2–4 min, 0→100% B; 4–8 min, 100% B; 8–9 min, 100→0% B; 9–10 min, 0% B
Thermo Scientific Hypersil Duet C18/SAX<br>4.6×250 mm, 5 µm	B1	A — 10 mmol L^{-1} ammonium acetate in water (pH=6.0), B — 10 mmol L^{-1} ammonium acetate in methanol (pH=6.0)<br>Gradient (1 mL min^{-1})<br>0–2 min, 0% B; 2–4 min, 0→100% B; 4–8 min, 100% B; 8–9 min, 100→0% B; 9–10 min, 0% B
Phenomenex PhenoSphere NEXT C18<br>4.6×250 mm, 5 µm	C1	A — 10 mmol L^{-1} ammonium acetate in water (pH=6.0), B — 10 mmol L^{-1} ammonium acetate in methanol (pH=6.0)<br>Gradient (1 mL min^{-1})<br>0–2 min, 0% B; 2–4 min, 0→100% B; 4–8 min, 100% B; 8–9 min, 100→0% B; 9–10 min, 0% B
Phenomenex HyperClone ODS (C18)<br>4.6×250 mm, 5 µm	D1	A — 10 mmol L^{-1} ammonium acetate in water (pH=6.0), B — 10 mmol L^{-1} ammonium acetate in methanol (pH=6.0)<br>Gradient (1 mL min^{-1})<br>0–2 min, 0% B; 2–4 min, 0→100% B; 4–13 min, 100% B; 13–15 min, 100→0% B
Shimadzu Shim-pack Scepter C8-120<br>4.6×150 mm, 3 µm	E1	A — 10 mmol L^{-1} ammonium acetate in water (pH=6.0), B — 10 mmol L^{-1} ammonium acetate in methanol (pH=6.0)<br>Gradient (1 mL min^{-1})<br>0–2 min, 0% B; 2–4 min, 0→100% B; 4–10 min, 100% B; 10–11 min, 100→0% B
	E2	A — 10 mmol L^{-1} ammonium acetate in water (pH=6.0), B — 10 mmol L^{-1} ammonium acetate in methanol (pH=6.0)<br>Gradient (0.2 mL min^{-1}) with after column dilution (0.6 mL min^{-1}, 1% nitric acid)<br>0–2 min, 0% B; 2–4 min, 0→100% B; 4–24 min, 100% B; 24–25 min, 100→0% B
	E3	A — 10 mmol L^{-1} ammonium acetate in water (pH=6.0), B — 10 mmol L^{-1} ammonium acetate in methanol (pH=6.0)<br>Gradient (0.2 mL min^{-1}) with after column dilution (0.6 mL min^{-1}, 1% nitric acid)<br>0–2 min, 0% B; 2–4 min, 0→100% B; 4–38 min, 100% B; 38–39 min, 100→0% B; 39–40 min, 0% B
	E4	A — 10 mmol L^{-1} ammonium acetate in water (pH=6.0), B — 10 mmol L^{-1} ammonium acetate in methanol (pH=6.0)<br>Gradient (0.2 mL min^{-1}) with after column dilution (0.6 mL min^{-1}, 1% nitric acid)<br>0–2 min, 0% B; 2–8 min, 0→100% B; 8–40 min, 100% B
	E5	A — 10 mmol L^{-1} ammonium acetate in water (pH=6.0), B — 10 mmol L^{-1} ammonium acetate in methanol (pH=6.0)<br>Gradient (0.2 mL min^{-1}) with after column dilution (0.6 mL min^{-1}, 1% nitric acid)<br>0–2 min, 0% B; 2–8 min, 0→100% B; 8–60 min, 100% B

	E6	B — 10 mmol L^{-1} ammonium acetate in methanol (pH=6.0)<br>Isocratic (0.2 mL min^{-1}) with after column dilution (0.6 mL min^{-1}, 1% nitric acid)<br>0–60 min, 100% B
Thermo Scientific Dionex IonPac CG5A<br>Shimadzu Shim-pack Scepter C8-120<br>4.6×150 mm, 3 µm	F1	A — 10 mmol L^{-1} ammonium acetate in water (pH=6.0), B — 10 mmol L^{-1} ammonium acetate in methanol (pH=6.0)<br>Gradient (0.2 mL min^{-1}) with after column dilution (0.7 mL min^{-1}, 1% nitric acid)<br>0–2 min, 0% B; 2–4 min, 0→100% B; 4–19 min, 100% B; 19–20 min, 100→0% B
	F2	A — 75 mmol L^{-1} ammonium nitrate in water (pH=9.0, 1% methanol), B — 10 mmol L^{-1} ammonium acetate in methanol (pH=6.0)<br>Gradient (0.2 mL min^{-1}) with after column dilution (0.7 mL min^{-1}, 1% nitric acid)<br>0–2 min, 0% B; 2–4 min, 0→100% B; 4–19 min, 100% B; 19–20 min, 100→0% B
	F3	A — 75 mmol L^{-1} ammonium nitrate in water (pH=9.0, 1% methanol), B — 10 mmol L^{-1} ammonium acetate in methanol (pH=6.0)<br>Gradient (0.2 mL min^{-1}) with after column dilution (0.7 mL min^{-1}, 1% nitric acid)<br>0–8 min, 0% B; 8–10 min, 0→100% B; 10–39 min, 100% B; 39–40 min, 100→0% B
Thermo Scientific Dionex IonPac CG5A<br>Shimadzu Shim-pack Scepter C18-120<br>4.6×150 mm, 3 µm	G1	A — 75 mmol L^{-1} ammonium nitrate in water (pH=9.0, 1% methanol), B — 10 mmol L^{-1} ammonium acetate in methanol (pH=6.0)<br>Gradient (0.2 mL min^{-1}) with after column dilution (0.7 mL min^{-1}, 1% nitric acid)<br>0–2 min, 0% B; 2–4 min, 0→100% B; 4–59 min, 100% B; 59–60 min, 100→0% B
	G2	A — 10 mmol L^{-1} ammonium acetate in water (pH=6.0), B — 10 mmol L^{-1} ammonium acetate in methanol (pH=6.0)<br>Gradient (0.2 mL min^{-1}) with after column dilution (0.7 mL min^{-1}, 1% nitric acid)<br>0–2 min, 0% B; 2–4 min, 0→100% B; 4–59 min, 100% B; 59–60 min, 100→0% B
Thermo Scientific Dionex IonPac CG5A<br>Thermo Scientific Dionex IonPac AS22<br>4.0×250 mm, 6 µm<br>Shimadzu Shim-pack Scepter C18-120<br>4.6×150 mm, 3 µm	H1	A — 10 mmol L^{-1} ammonium acetate in water (pH=6.0), B — 10 mmol L^{-1} ammonium acetate in methanol (pH=6.0)<br>Gradient (0.4 mL min-1) with after column dilution (0.7 mL min-1, 1% nitric acid)<br>0–4 min, 0% B; 4–6 min, 0→100% B; 6–39 min, 100% B; 39–40 min, 100→0% B; 40–41 min, 0% B
	H2	A — 20 mmol L^{-1} ammonium acetate in water (pH=9.2), B — 10 mmol L^{-1} ammonium acetate in methanol (pH=6.0)<br>Gradient (0.4 mL min-1) with after column dilution (0.7 mL min-1, 1% nitric acid)<br>0–4 min, 0% B; 4–6 min, 0→100% B; 6–39 min, 100% B; 39–40 min, 100→0% B; 40–41 min, 0% B
	H3	A — 20 mmol L^{-1} ammonium acetate in water (pH=9.2), B — 10 mmol L^{-1} ammonium acetate in methanol (pH=6.0)<br>Gradient (0.4 mL min-1) with after column dilution (0.7 mL min-1, 1% nitric acid)<br>0–15 min, 0% B; 15–20 min, 0→100% B; 20–60 min, 100% B; 60–61 min, 100→0% B

	H4	A — 10 mmol L^{-1} ammonium acetate in water (pH=6.0), B — 10 mmol L^{-1} ammonium acetate in methanol (pH=6.0)<br>Gradient (0.4 mL min^{-1}) with after column dilution (0.7 mL min^{-1}, 1% nitric acid)<br>0–15 min, 0% B; 15–20 min, 0→100% B; 20–60 min, 100% B; 60–61 min, 100→0% B
	H5	A — 10 mmol L^{-1} ammonium acetate in water (pH=6.0), B — 20 mmol L^{-1} ammonium acetate in methanol (pH=9.2)<br>Gradient (0.4 mL min^{-1}) with after column dilution (0.7 mL min^{-1}, 1% nitric acid)<br>0–15 min, 0% B; 15–20 min, 0→100% B; 20–60 min, 100% B; 60–61 min, 100→0% B
Shimadzu Shim-pack Scepter C18-120<br>4.6×150 mm, 3 µm<br>Thermo Scientific Dionex IonPac AS22<br>4.0×250 mm, 6 µm<br>Thermo Scientific Dionex IonPac CG5A	I1	A — 10 mmol L^{-1} ammonium acetate in water (pH=6.0), B — 10 mmol L^{-1} ammonium acetate in methanol (pH=6.0)<br>Gradient (0.4 mL min^{-1}) with after column dilution (0.7 mL min^{-1}, 1% nitric acid)<br>0–15 min, 0% B; 15–20 min, 0→100% B; 20–60 min, 100% B; 60–61 min, 100→0% B
Shimadzu Shim-pack Scepter C18-120<br>4.6×150 mm, 3 µm<br>Hamilton PRP-X110<br>4.6×250 mm, 7 µm	J1	A — 10 mmol L^{-1} ammonium acetate in water (pH=6.0), B — 10 mmol L^{-1} ammonium acetate in methanol (pH=6.0)<br>Gradient (0.4 mL min^{-1}) with after column dilution (0.7 mL min^{-1}, 1% nitric acid)<br>0–15 min, 0% B; 15–20 min, 0→100% B; 20–60 min, 100% B; 60–61 min, 100→0% B
	J2	A — 1 mmol L^{-1} ammonium acetate in water (pH=6.0), B — 1 mmol L^{-1} ammonium acetate in methanol (pH=6.0)<br>Gradient (0.4 mL min^{-1}) with after column dilution (0.7 mL min^{-1}, 1% nitric acid)<br>0–15 min, 0% B; 15–20 min, 0→100% B; 20–60 min, 100% B; 60–61 min, 100→0% B
	J3	A — 1 mmol L^{-1} ammonium acetate in water (pH=6.0), B — 10 mmol L^{-1} ammonium acetate in methanol (pH=6.0)<br>Gradient (0.4 mL min^{-1}) with after column dilution (0.7 mL min^{-1}, 1% nitric acid)<br>0–15 min, 0% B; 15–20 min, 0→100% B; 20–60 min, 100% B; 60–61 min, 100→0% B
	J4	A — 1 mmol L^{-1} ammonium acetate in water (pH=9.2), B — 20 mmol L^{-1} ammonium acetate in methanol (pH=9.2)<br>Gradient (0.4 mL min^{-1}) with after column dilution (0.7 mL min^{-1}, 1% nitric acid)<br>0–15 min, 0% B; 15–20 min, 0→100% B; 20–60 min, 100% B; 60–61 min, 100→0% B

Table S7. Retention times [min] and peak areas of arsenic species in mixed standard solutions (1 mg L^{-1}).

Analysis conditions*	As(III), As(V)		As(III), As(V), AsB, DMA		AsFA 362		AsHC 360	
	Retention time	Peak area	Retention time	Peak area	Retention time	Peak area	Retention time	Peak area
G2	10.452	1730.695			13.223	1486.610	40.923	1219.842
H1	12.651	1371.365			14.453	1238.568	29.416	911.459
H2	11.279	1674.136			26.139	1075.984	34.250	709.359
H3			11.959	3273.392	36.284	917.852	57.646	1123.923
H4			11.033	3117.525	40.701	1120.400	59.641	645.025
H5			10.678	3369.285	41.982	1076.932	50.543	930.355
I1	14.164	1027.162			37.825	829.202	53.725	623.990
J1	14.705	936.154			43.606	1002.203	53.073	827.665
J2	14.674	1068.379			-	-	-	-
J3	11.839	1209.206			57.807	1097.922	-	-
J4	11.911	2219.876			49.862	1151.331	52.890	1078.078

* The analysis conditions are explained in Table S6.

Arsenolipids synthesis

Iododimethylarsine synthesis

In a round-bottom flask flask sealed with a rubber septa, DMA (2.0 g), KI (6.6 g) and $NaHSO_3$ (1.0 g) were dissolved in freshly deionized water (8 mL) under magnetic stirring until all reagents were fully dissolved and the solution color changed from colorless to yellow (~5 min). Subsequently, concentrated HCl (10 mL) was rapidly added via syringe and needle to the continuously stirred reaction mixture, causing an immediate color change to brownish-orange and the formation of a dark layer at the bottom of the flask. The reaction mixture was then stirred at room temperature overnight (22 h). After stirring was stopped, three distinct layers were observed: a dark bottom layer, a light intermediate layer, and an orange upper solution. The entire contents of the flask were transferred to a glass separatory funnel. The flask was rinsed several times with freshly deionized water, and the rinses were combined in the funnel to a total aqueous volume of 60 mL. The aqueous phase was extracted with chloroform: once with 20 mL and then twice with 40 mL. The chloroform extracts were combined, dried over Na_2SO_4, and concentrated using a rotary evaporator to yield a brownish-yellow oil. The synthesis of iododimethylarsine was also successfully performed on a twofold scale using doubled reagent quantities. Additionally, to ensure the purity of the synthesized iododimethylarsine, the crude product was distilled under reduced pressure and collected as a yellow oil (72 °C, 90 mbar). Iododimethylarsine was obtained in 41.2% yield (11.1 g). The purified product was stored in a refrigerator.

Bis(dimethylarsenic) oxide synthesis

Under an argon atmosphere, a two-neck round-bottom flask equipped with a condenser, both sealed with rubber septa, was placed in an ice bath on a magnetic stirrer. A NaOH solution (10 M, 5.0 mL) was introduced into the flask, stirred, and cooled. Subsequently, iododimethylarsine (0.5 mL) was added to the stirred solution via syringe and needle. The yellow color of iododimethylarsine disappeared immediately, yielding a colorless reaction mixtures with clear oil drops forming on the surface. The mixture was stirred for 15 minutes, during which an emulsion developed. Immediately afterward, the resulting bis(dimethylarsenic) oxide was used — within the same reaction setup — for the synthesis of selected arsenolipids.

15-(Dimethylarsinyl)pentadecanoic, AsFA 362, synthesis

Under an argon atmosphere, the freshly synthesized bis(dimethylarsenic) oxide, prepared according to the procedure described in Section 2.2.2 and kept under magnetic stirring, was treated with a freshly prepared suspension of 15-bromopentadecanoic acid (1.6 g) in ethanol (10 mL). During the addition of the suspension, white gas was evolved and the white mixture thickened slightly. The reaction setup was then placed in a heated oil bath (80 °C) and stirred overnight. Immediately after immersion in the oil bath, the reaction mixture became clear, and within a few minutes turned white again. Continued heating caused gradual yellowing and darkening of the mixture until it reached a dark brownish-orange color. The flask was removed from the oil bath and the reaction mixture was allowed to cool to room temperature. The contents were transferred to a separatory funnel, freshly deionized water was added to a final volume of 40 mL, and the aqueous layer was extracted three times with diethyl ether (40 mL each). During extraction, the aqueous layer exhibited a light brownish-orange color, while the

ether layer changed from yellow after the first extraction to clear after the third. The pH of the aqueous layer was then adjusted to 3.5 using HCl (6 M, a few drops). The aqueous layer was extracted with chloroform (3 × 40 mL). The combined chloroform extracts were washed with freshly deionized water (3 × 120 mL), dried over Na_2SO_4, and concentrated on a rotary evaporator. During evaporation, the color changed from a light orangish-yellow liquid to a reddish-orange residue. The residue was purified by crystallization from ethyl acetate, followed by filtration under pressure, yielding the product as a white solid with a yield of 44.1% (0.8 g).

1-(Dimethylarsinyl)heptadecane, AsHC 360, synthesis

The synthesis of AsHC 360 was carried out following the same procedure as that described for AsFA 362, with minor modifications. A suspension of 1-bromoheptadecane (1.6 g) in ethanol (10 mL) was used, and the pH was adjusted to 7 rather than to 3.5, as in the case of AsFA 362. The product was obtained as a white solid in 22.2% yield (0.4 g).

Additional observations

The described syntheses were conducted based on the reported procedures [64, 74], with changes acknowledging available laboratory equipment and capabilities (Tables S3–S5). Our aim was not necessarily to improve the existing synthesis methods, but rather to reproduce them in our laboratory. It should be noted that we obtained the selected arsenolipids in lower yields than those reported in the literature. The detailed description of the syntheses performed, including the issues encountered and their solutions, enables their reproduction. The issues are summarized below.

The first issue was encountered during the synthesis of iododimethylarsine. The product was contaminated, as a dark reddish-brown oil was obtained instead of the expected yellow oil, and the evaporated chloroform appeared red rather than clear. This problem was resolved in subsequent syntheses by vigorously shaking the separatory funnel during the water and chloroform extraction, which resulted in a white aqueous layer and a light-yellow chloroform layer. Initially, the extraction had been performed by gently mixing the funnel contents, which proved insufficient for proper separation. The second issue arose during the synthesis of AsHC 360, where the product was observed to partition into the diethyl ether layer, instead of remaining in the aqueous layer as intended. This behavior could have been caused by a low reaction yield and interactions between unreacted starting materials and the product. To address this, the diethyl ether layer was first evaporated using a rotatory evaporator, and the sample of the residue was collected for a LC-MS analysis to confirm the presence of the product. The extraction was then repeated using a modified diethyl ether-to-water ratio of 1:2, with 90 mL of freshly deionized water and 45 mL of diethyl ether. However, the layers did not separate immediately, and the mixture had to be left in the refrigerator. After one week, the layers separated, and extraction of the aqueous layer was continued according to the procedure described in Section 2.2.4, starting with pH adjustment to 7. Additionally, the synthesis of AsFA 362 was conducted following the suggestion of Chacon-Teran et al. [73], regarding neat conditions for the generation of bis(dimethylarsenic) oxide. However, this approach proved more challenging than the procedure described in Section 2.2.3, and the product was obtained in a lower yield (34.4%). Following the observation reported by Chacon-Teran et al. [73], neat NaOH pellets were used instead of an aqueous NaOH solution (10 M, 5 mL). Under an argon atmosphere, in a two-neck round-bottom flask equipped with a condenser, both sealed with rubber septa and placed in an ice bath on a magnetic stirrer, dimethyliodoarsine (0.5 mL, 1.5 g,

6.3 mmol) was added via syringe and cooled to 2 °C while stirring. To this, neat NaOH (250.6 mg, 6.3 mmol) was added. It is important to note that this approach required opening the reaction setup, posing a risk of exposure to toxic dimethyliodoarsine and bis(dimethylarsenic) oxide, which should be avoided. As reported by Chacon-Teran et al. [73], the formation of bis(dimethylarsenic) oxide is indicated by the mixture turning colorless, however, our mixture remained light yellow, so additional NaOH (178.3 mg, 4.5 mmol) was added. The mixture gradually lightened during stirring, but almost dried out. Therefore, a suspension of 15-bromopentadecanoic acid (335.5 mg, 1.0 mmol) in 2 mL of ethanol was added portion-wise via syringe during which a white gas was released. The reaction setup was then placed in a heated oil bath (80 °C) and stirred overnight, during which the mixture darkened to a brownish-orange color. The subsequent work-up of AsFA 362 followed the same procedure as described. However, under this synthesis approach, the water/ethyl ether extraction was less efficient, and layer separation during each extraction required at least 20 minutes, exceeding 30 minutes during the third and final extraction.

Method development

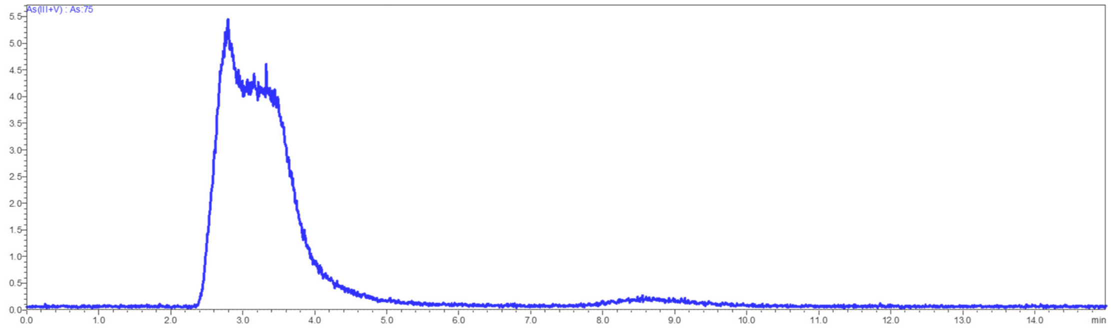

Figure S1. Chromatogram of the mix of As(III) and As(V), 10 µg L^{-1}, prepared in mobile phase A (10 mmol L^{-1} ammonium acetate in water, pH=6.0).
Gradient (1 mL min^{-1}): 0–2 min, 0% B; 2–4 min, 0→100% B; 4–13 min, 100% B; 13–15 min, 100→0% B.
Column: Phenomenex HyperClone ODS (C18) (4.6×250 mm, 5 µm). Injection volume: 100 µL.

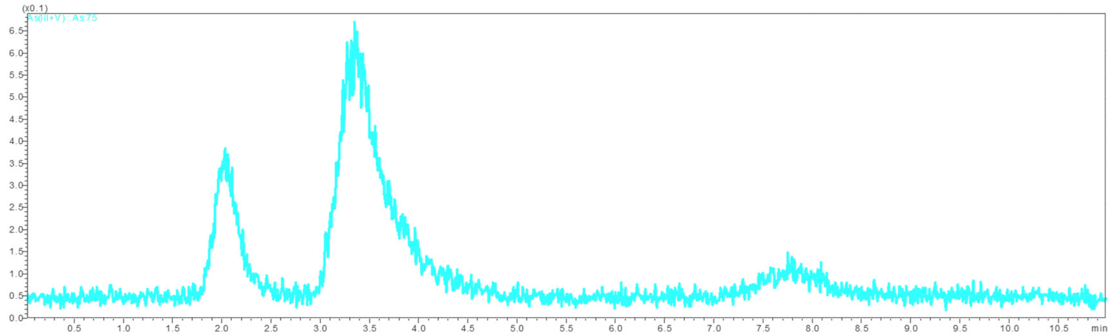

Figure S2. Chromatogram of the mix of As(III) and As(V), 10 µg L^{-1}, prepared in the mobile phase A (10 mmol L^{-1} ammonium acetate in water, pH=6.0).
Gradient (1 mL min^{-1}): 0–2 min, 0% B; 2–4 min, 0→100% B; 4–10 min, 100% B; 10–11 min, 100→0% B.
Column: Shimadzu Shim-pack Scepter C8-120 (4.6×150 mm, 3 µm). Injection volume: 100 µL.

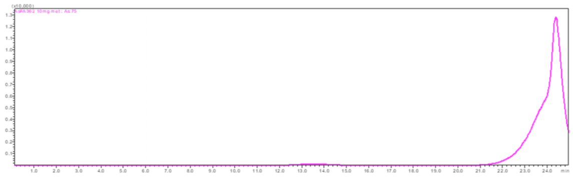

(a) Injection of AsFA 362, 10 mg L^{-1}

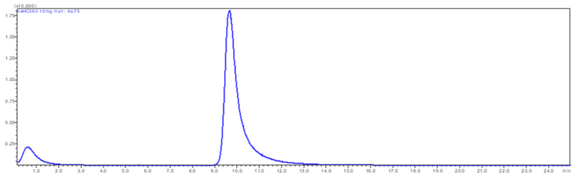

(b) Injection of AsHC 360, 10 mg L^{-1}

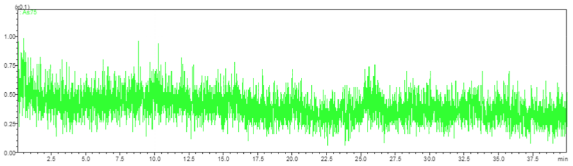

(c) Mobile phase

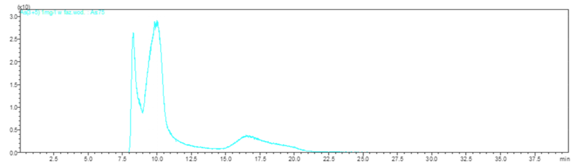

(d) As(III) + As(V), 1 mg L^{-1}; t_{R1} = 8.284, t_{R2} = 9.999

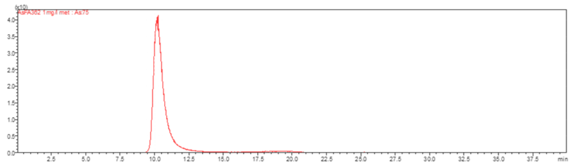

(e) AsFA 362, 1 mg L^{-1}; t_R = 10.276

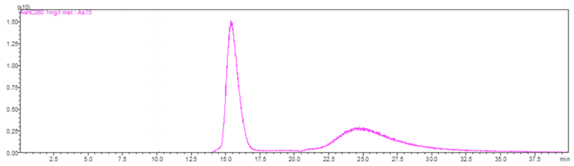

(f) AsHC 360, 1 mg L^{-1}; t_{R1} = 15.385, t_{R2} = 24.750

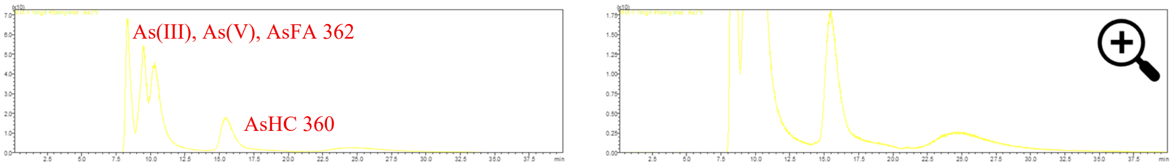

(g) MIX (1 mg L^{-1}): As(III), As(V), AsFA 362, and AsHC 360; t_{R1} = 8.291, t_{R2} = 9.460, t_{R3} = 10.295, t_{R4} = 15.512, t_{R5} = 24.786

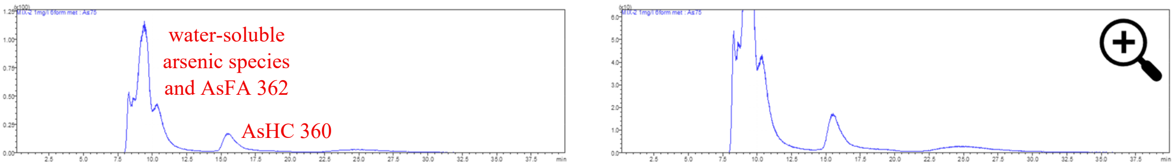

(h) MIX (1 mg L^{-1}): As(III), As(V), AsB, DMA, AsFA 362, and AsHC 360; t_{R1} = 8.305, t_{R2} = 8. 610, t_{R3} = 9.428, t_{R4} = 10.344, t_{R5} = 15.519, t_{R6} = 24.837

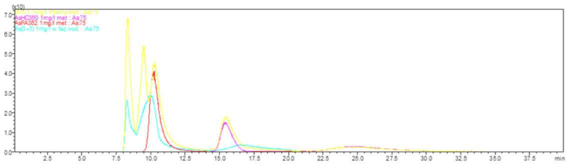

(i) Overlaid chromatograms: d–g

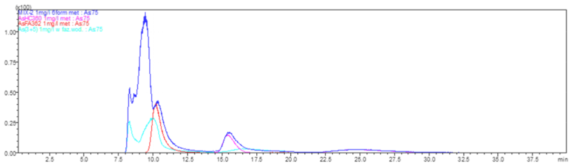

(j) Overlaid chromatograms: d–f and h

Figure S3. Chromatograms (a, b, e, f) of the synthesized AsFA 362 and AsHC 360, 1 mg L^{-1}, prepared in methanol; chromatogram (c) of the mobile phase; chromatogram (d) of the mix of As(III) and As(V), 1 mg L^{-1}, prepared in the mobile phase A (10 mmol L^{-1} ammonium acetate in water, pH=6.0); chromatogram (g) of the mix of 4 arsenic species — As(III), As(V), AsFA 362 and AsHC 360, 1 mg L^{-1}, prepared in methanol; chromatogram (h) of the mix of 6 arsenic species — As(III), As(V), AsB, DMA, AsFA 362 and AsHC 360, 1 mg L^{-1}, prepared in methanol; and overlaid chromatograms (i, j).
Gradient (0.2 mL min^{-1}) with after column dilution (0.6 mL min^{-1}, 1% nitric acid): (a–b): 0–2 min, 0% B; 2–4 min, 0→100% B; 4–24 min, 100% B; 24–25 min, 100→0% B, (c–j): 0–2 min, 0% B; 2–4 min, 0→100% B; 4–38 min, 100% B; 38–39 min, 100→0% B; 39–40 min, 0% B. Column: Shimadzu Shim-pack Scepter C8-120 (4.6×150 mm, 3 µm). Injection volume: 10 µL.

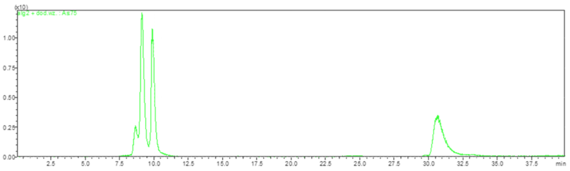

(a) Injection of sample (marine algae)

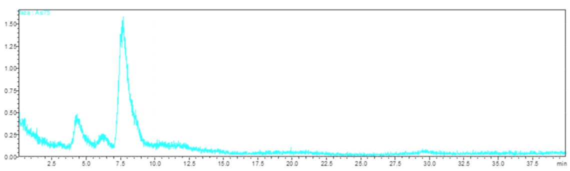

(b) Injection of mobile phase

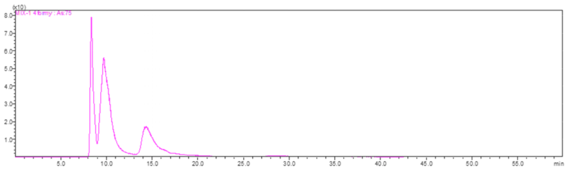

(c) MIX (1 mg L^{-1}): As(III), As(V), AsFA 362, and AsHC 360; t_{R1} = 8.348, t_{R2} = 9.708, t_{R3} = 14.361

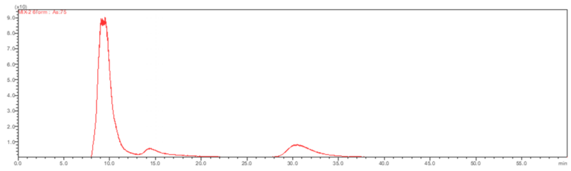

(d) MIX (1 mg L^{-1}): As(III), As(V), AsB, DMA, AsFA 362, and AsHC 360; t_{R1} = 9.501, t_{R2} = 14.335, t_{R3} = 30.329

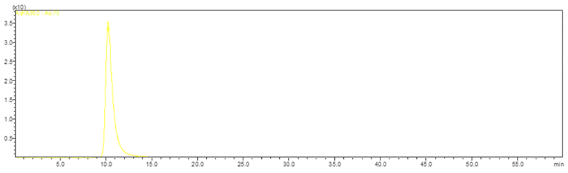

(e) AsFA 362, 1 mg L^{-1}, t_R = 10.225

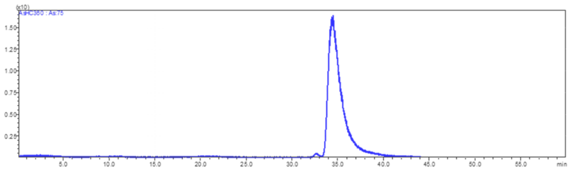

(f) AsHC 360, 1 mg L^{-1}, t_R = 34.491

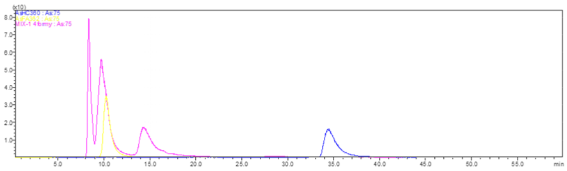

(g) Overlaid chromatograms: c, e and f

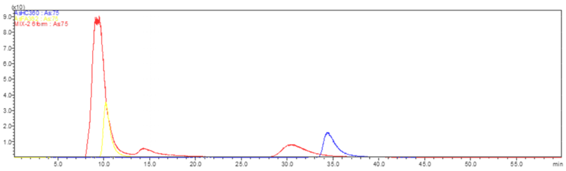

(h) Overlaid chromatograms: d–f

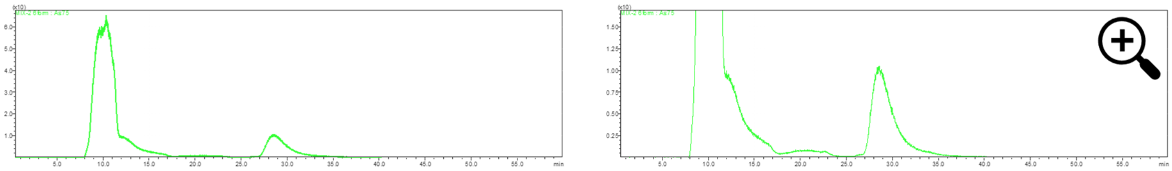

(i) MIX (1 mg L^{-1}): As(III), As(V), AsB, DMA, AsFA 362, and AsHC 360; t_{R1} = 10.325, t_{R2} = 11.960, t_{R3} = 20.933, t_{R4} = 28.584

Figure S4. Chromatogram (a) of the sample — the marine algae sample with the addition of arsenolipids standards, extracted with methanol; chromatogram (b) of the mobile phase; chromatogram (c) of the mix of 4 arsenic species — As(III), As(V), AsFA 362 and AsHC 360, 1 mg L^{-1}, prepared in methanol; chromatograms (d, i) of the mix of 6 arsenic species — As(III), As(V), AsB, DMA, AsFA 362 and AsHC 360, 1 mg L^{-1}, prepared in methanol; chromatograms (e, f) of the synthesized AsFA 362 and AsHC 360, 1 mg L^{-1}, prepared in methanol; and overlaid chromatograms (g, h). *There are arsenic peaks, from the previous analysis of the marine algae, visible at the beginning of the chromatogram b, showing that the analysis was too short.*
Gradient (0.2 mL min^{-1}) with after column dilution (0.6 mL min^{-1}, 1% nitric acid): (a–b) 0–2 min, 0% B; 2–8 min, 0→100% B; 8–40 min, 100% B, (c–h) 0–2 min, 0% B; 2–8 min, 0→100% B; 8–60 min, 100% B, (i) 0–60 min, 100% B. Column: Shimadzu Shim-pack Scepter C8-120 (4.6×150 mm, 3 µm). Injection volume: 10 µL.

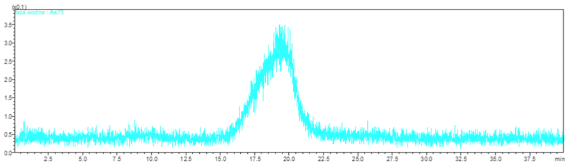

(a) Mobile phase

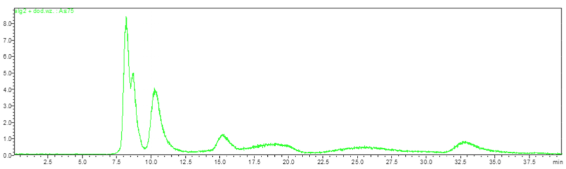

(b) Marine algae with addition of arsenolipids standards; t_{R1} = 8.189, t_{R2} = 8.678, t_{R3} = 10.251, t_{R4} = 15.201, t_{R5} = 18.710, t_{R6} = 25.519, t_{R7} = 32.709

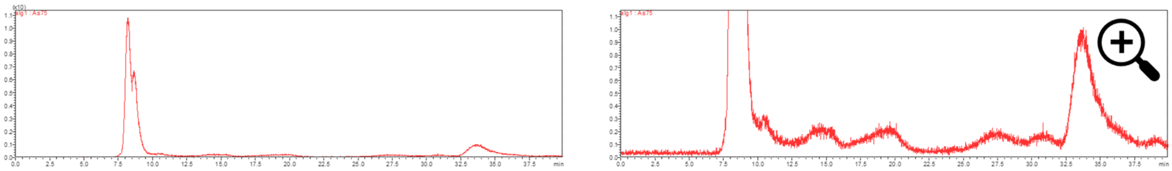

(c) Marine algae; t_{R1} = 8.181, t_{R2} = 8.654, t_{R3} = 10.420, t_{R4} = 14.491, t_{R5} = 19.620, t_{R6} = 27.109, t_{R7} = 30.820, t_{R8} = 33.761, t_{R9} = 38.935

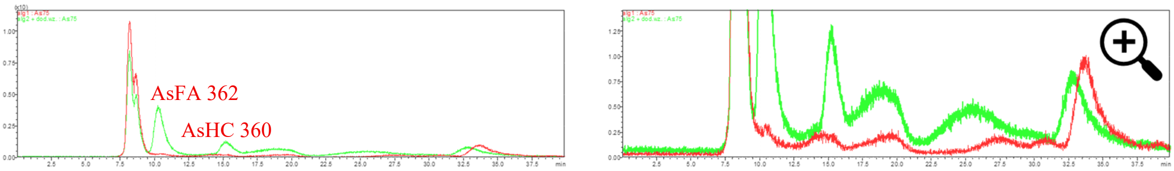

(d) Overlaid chromatograms: b and c

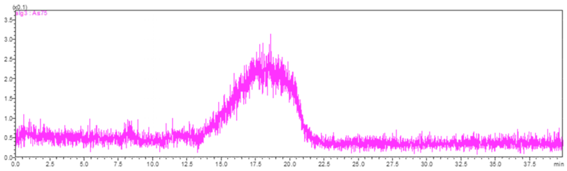

(e) Red algae; t_R = 18.618

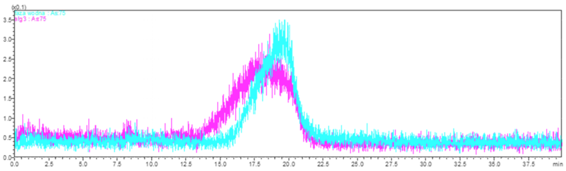

(f) Red algae and mobile phase

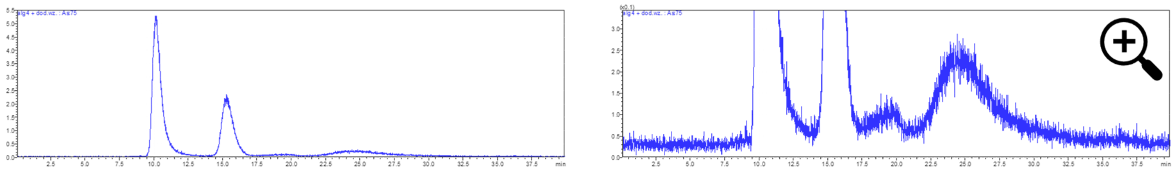

(g) Red algae with addition of arsenolipids standards; $t_{R1} = 10.134$, $t_{R2} = 15.285$, $t_{R3} = 18.878$, $t_{R4} = 24.415$

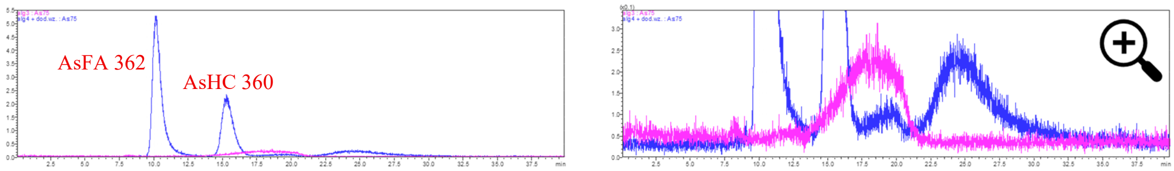

(h) Overlaid chromatograms: e and g

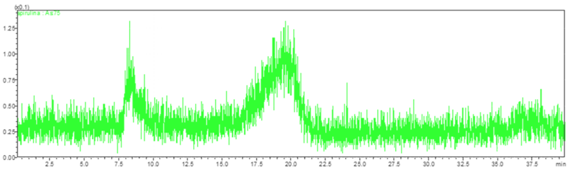

(i) Spirulina; $t_{R1} = 8.291$, $t_{R2} = 19.601$

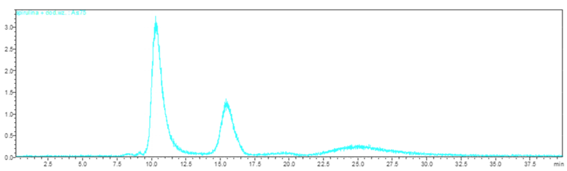

(j) Spirulina with addition of arsenolipids standards; $t_{R1} = 8.224$, $t_{R2} = 9.167$, $t_{R3} = 10.318$, $t_{R4} = 15.430$, $t_{R5} = 19.796$, $t_{R6} = 25.124$

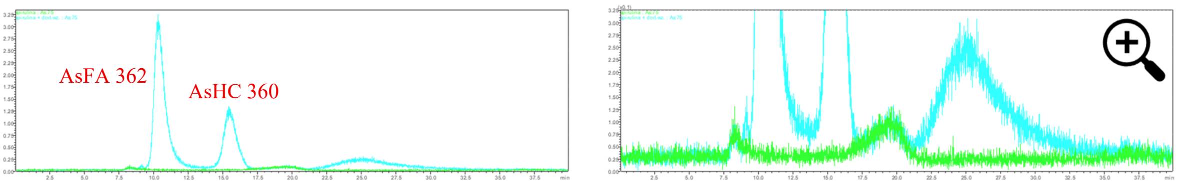

(k) Overlaid chromatograms: i and j

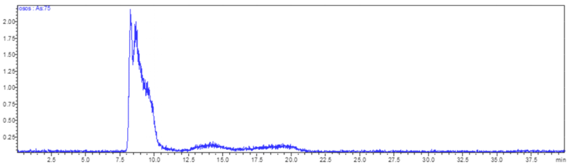

(l) Salmon; $t_{R1} = 8.273$, $t_{R2} = 8.696$, $t_{R3} = 9.198$, $t_{R4} = 14.299$, $t_{R5} = 19.389$

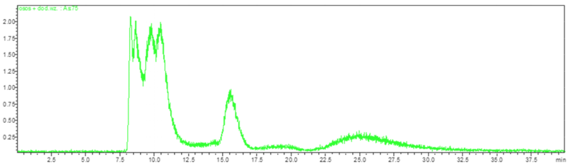

(m) Salmon with addition of arsenolipids standards; $t_{R1} = 8.280$, $t_{R2} = 8.660$, $t_{R3} = 9.813$, $t_{R4} = 10.472$, $t_{R5} = 15.603$, $t_{R6} = 19.274$, $t_{R7} = 24.955$

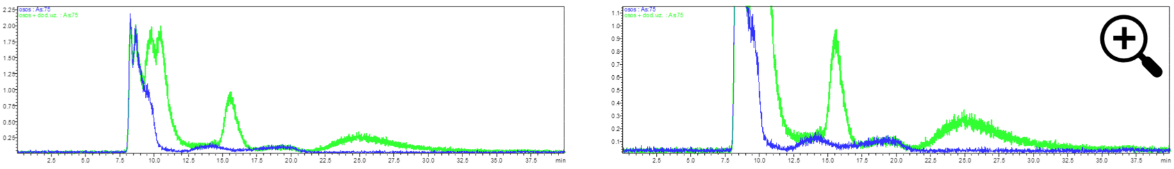

(n) Overlaid chromatograms: l and m

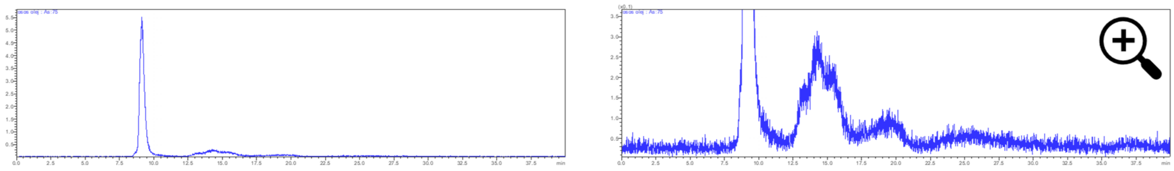

(o) Salmon–oil; $t_{R1} = 9.118$, $t_{R2} = 13.316$, $t_{R3} = 14.249$, $t_{R4} = 15.150$, $t_{R5} = 19.512$, $t_{R6} = 23.838$

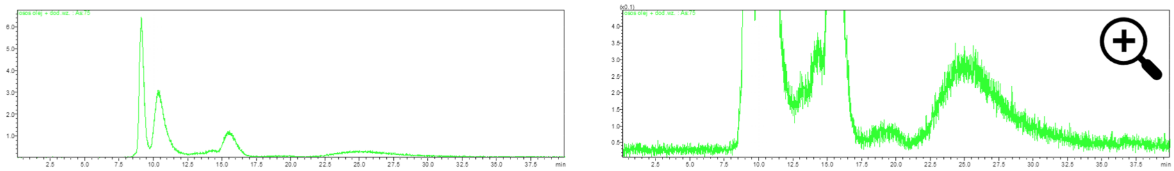

(p) Salmon–oil with addition of arsenolipids standards; $t_{R1} = 9.100$, $t_{R2} = 10.351$, $t_{R3} = 13.029$, $t_{R4} = 14.297$, $t_{R5} = 15.556$, $t_{R6} = 19.138$, $t_{R7} = 24.348$

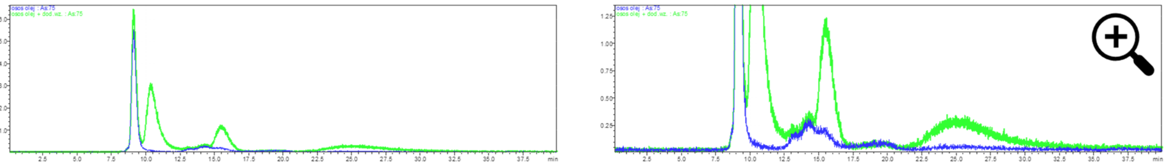

(r) Overlaid chromatograms: o and p

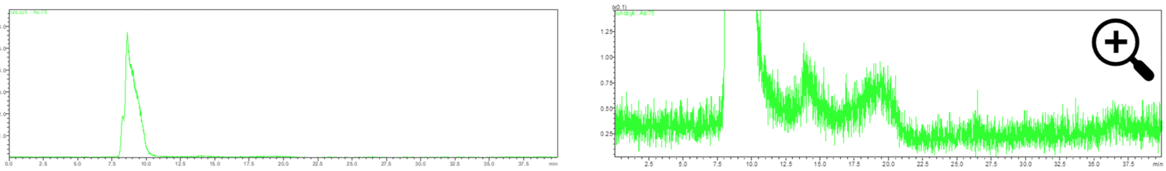

(s) Tuna; t_{R1} = 8.284, t_{R2} = 8.597, t_{R3} = 13.819, t_{R4} = 19.391

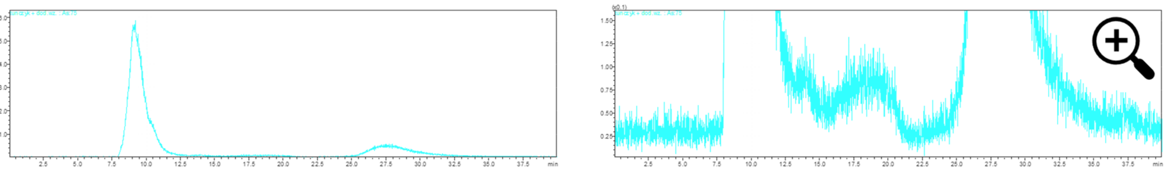

(t) Tuna with addition of arsenolipids standards; t_{R1} = 9.227, t_{R2} = 13.938, t_{R3} = 17.127, t_{R4} = 27.944

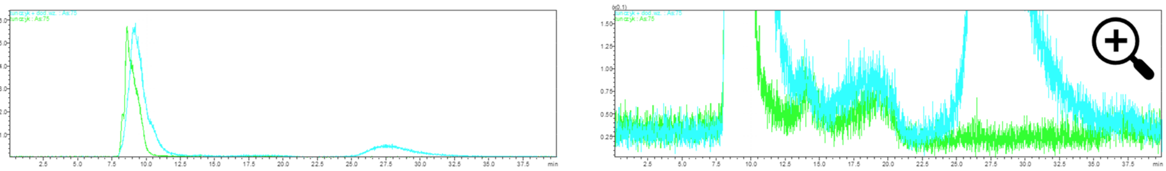

(u) Overlaid chromatograms: s and t

Figure S5. Chromatogram (a) of the mobile phase; chromatograms (b, c) of the marine algae sample without and with the addition of arsenolipids standards, extracted with methanol; chromatograms (e, g) of the red algae sample without and with the addition of arsenolipids standards, extracted with methanol; chromatograms (i, j) of the spirulina sample without and with the addition of arsenolipids standards, extracted with methanol; chromatograms (l, m) of the salmon sample without and with the addition of arsenolipids standards, extracted with methanol/water; chromatograms (o, p) of the salmon–oil, which separated during the salmon sample preparation, without and with the addition of arsenolipids standards; chromatograms (s, t) of the tuna sample without and with the addition of arsenolipids standards, extracted with methanol/water; and overlaid chromatograms (d, f, h, k, n, r, u).

Gradient (0.2 mL min^{-1}) with after column dilution (0.6 mL min^{-1}, 1% nitric acid): 0–2 min, 0% B; 2–4 min, 0→100% B; 4–38 min, 100% B; 38–39 min, 100→0% B; 39–40 min, 0% B. Column: Shimadzu Shim-pack Scepter C8-120 (4.6×150 mm, 3 µm). Injection volume: 10 µL.

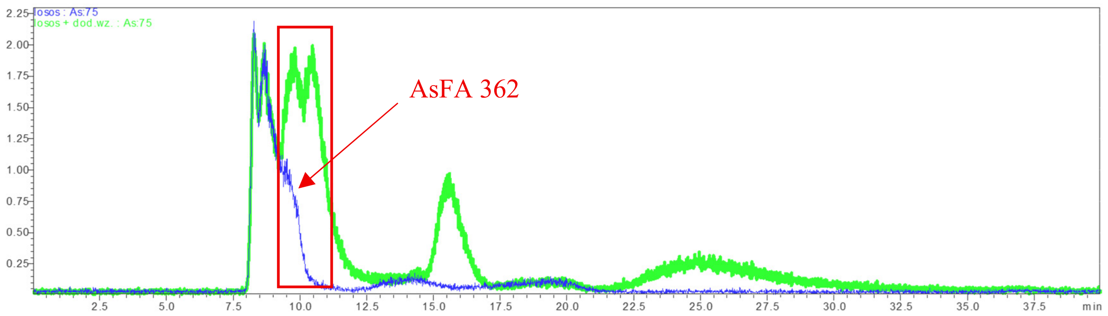

(a) Salmon without (blue) and with (green) addition of arsenolipids standards

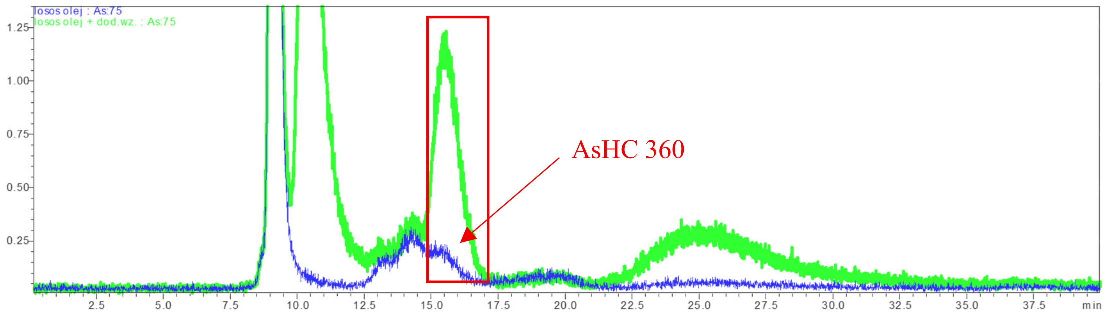

(b) Salmon–oil without (blue) and with (green) addition of arsenolipids standards

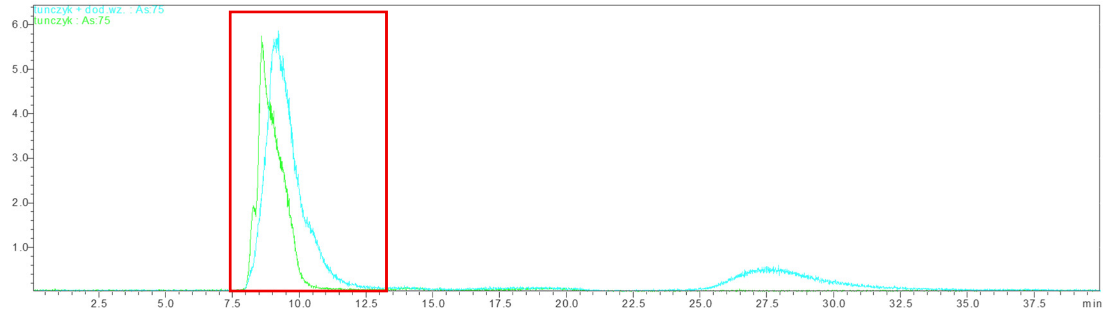

(c) Tuna without (green) and with (blue) addition of arsenolipids standards

Figure S6. Comparison of the chromatograms obtained for the fish samples without and with the addition of the arsenolipids standards – AsFA 362 and AsHC 360.

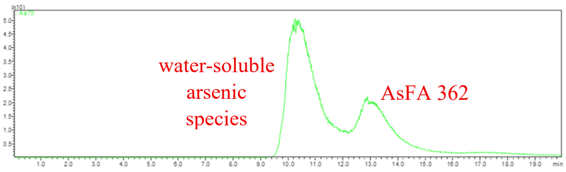

(a) MIX (1 mg L^{-1}): As(III), As(V), AsB, DMA, AsFA 362, and AsHC 360; t_{R1} = 10.257, t_{R2} = 12.902

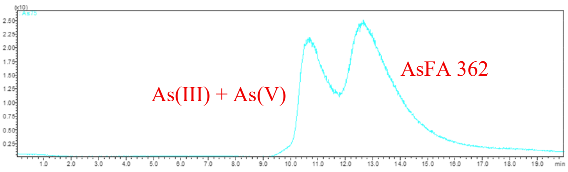

(b) MIX (1 mg L^{-1}): As(III), As(V), AsFA 362, and AsHC 360; t_{R1} = 10.698, t_{R2} = 12.658

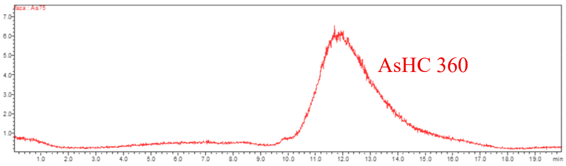

(c) Injection of mobile phase

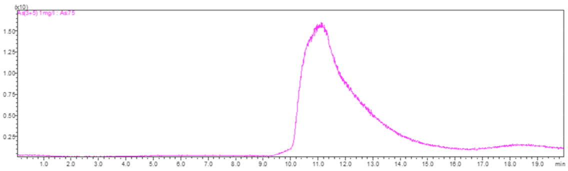

(d) As(III) + As(V), 1 mg L^{-1}; t_R = 11.116

Figure S7. Chromatogram (a) of the mix of 6 arsenic species — As(III), As(V), AsB, DMA, AsFA 362 and AsHC 360, 1 mg L^{-1}, prepared in methanol; chromatogram (b) of the mix of 4 arsenic species — As(III), As(V), AsFA 362 and AsHC 360, 1 mg L^{-1}, prepared in methanol; chromatogram (c) of the mobile phase; and chromatogram (d) of the mix of As(III) and As(V), 1 mg L^{-1}, prepared in the mobile phase A (10 mmol L^{-1} ammonium acetate in water, pH=6.0). *The AsHC 360 peak, which was not eluted during the analysis of the mix of 4 arsenic species, is visible in the chromatogram c, showing that the analysis was too short.*
Gradient (0.2 mL min^{-1}) with after column dilution (0.7 mL min^{-1}, 1% nitric acid): 0–2 min, 0% B; 2–4 min, 0→100% B; 4–19 min, 100% B; 19–20 min, 100→0% B. Columns: Thermo Scientific Dionex IonPac CG5A and Shimadzu Shim-pack Scepter C8-120 (4.6×150 mm, 3 µm). Injection volume: 10 µL.

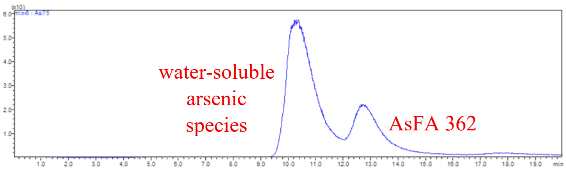

(a) MIX (1 mg L^{-1}): As(III), As(V), AsB, DMA, AsFA 362, and AsHC 360; t_{R1} = 10.220, t_{R2} = 12.691

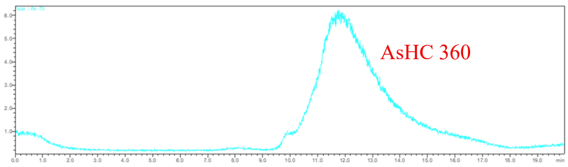

(b) Mobile phase

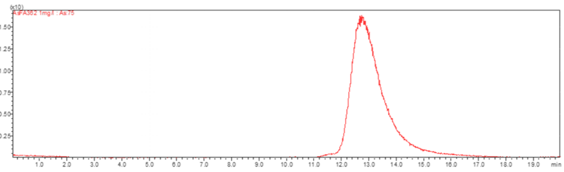

(c) AsFA 362, 1 mg L^{-1}; t_R = 12.733

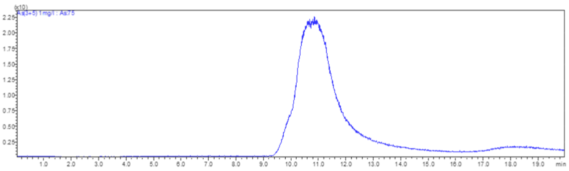

(d) As(III) + As(V), 1 mg L^{-1}; t_R = 10.859

Figure S8. Chromatogram (a) of the mix of 6 arsenic species — As(III), As(V), AsB, DMA, AsFA 362 and AsHC 360, 1 mg L^{-1}, prepared in methanol; chromatogram (b) of the mobile phase; chromatogram (c) of the synthesized AsFA 362, 1 mg L^{-1}, prepared in methanol; and chromatogram (d) of the mix of As(III) and As(V), 1 mg L^{-1}, prepared in the mobile phase A (10 mmol L^{-1} ammonium acetate in water, pH=6.0). *The AsHC 360 peak, which was not eluted during the analysis of the mix of 6 arsenic species, is visible in the chromatogram b, showing that the analysis was too short.*
Gradient (0.2 mL min^{-1}) with after column dilution (0.7 mL min^{-1}, 1% nitric acid): 0–2 min, 0% B; 2–4 min, 0→100% B; 4–19 min, 100% B; 19–20 min, 100→0% B. New mobile phase A: 75 mmol L^{-1} ammonium nitrate in water (pH=9.0, 1% methanol). Columns: Thermo Scientific Dionex IonPac CG5A and Shimadzu Shim-pack Scepter C8-120 (4.6×150 mm, 3 µm). Injection volume: 10 µL.

.

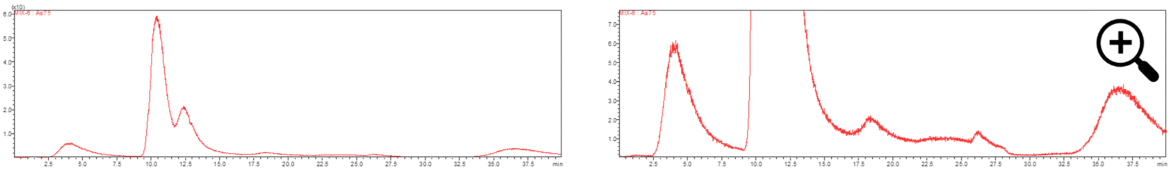

(a) MIX (1 mg L^{-1}): As(III), As(V), AsB, DMA, AsFA 362, and AsHC 360; t_{R1} = 4.152, t_{R2} = 10.396, t_{R3} = 12.394, t_{R4} = 18.383, t_{R5} = 26.210, t_{R6} = 36.839

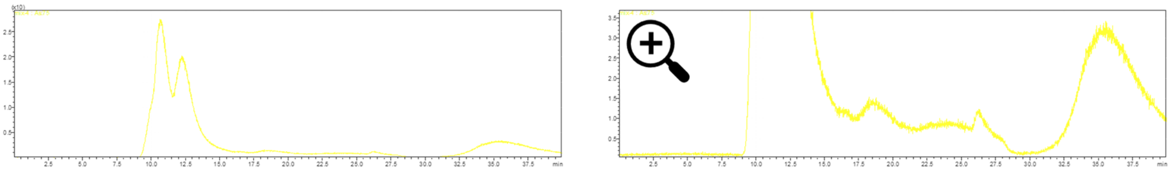

(b) MIX (1 mg L^{-1}): As(III), As(V), AsFA 362, and AsHC 360; t_{R1} = 10.639, t_{R2} = 12.242, t_{R3} = 18.287, t_{R4} = 26.251, t_{R5} = 35.554

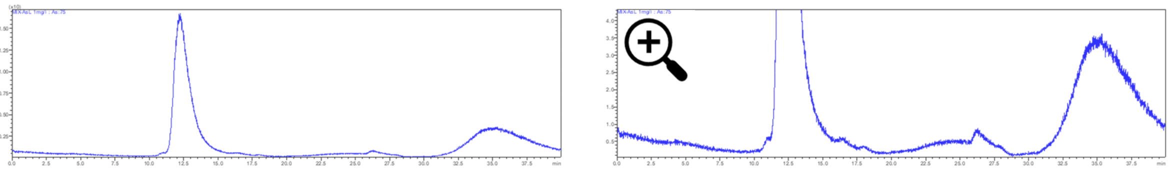

(c) AsFA 362 + AsHC 360, 1 mg L^{-1}; t_{R1} = 12.231, t_{R2} = 16.210, t_{R3} = 17.936, t_{R4} = 26.296, t_{R5} = 35.292

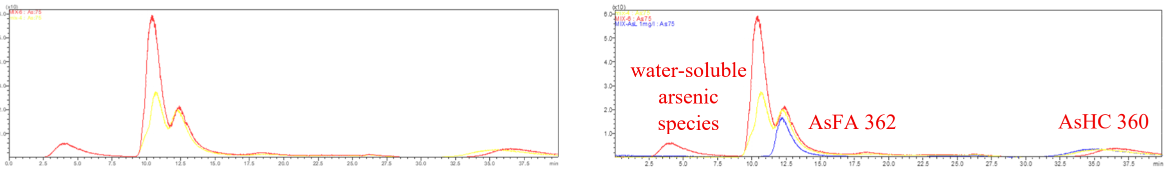

(d) Overlaid chromatograms: a and b (e) Overlaid chromatograms: a–c

Figure S9. Chromatogram (a) of the mix of 6 arsenic species — As(III), As(V), AsB, DMA, AsFA 362 and AsHC 360, 1 mg L^{-1}, prepared in methanol; chromatogram (b) of the mix of 4 arsenic species — As(III), As(V), AsFA 362 and AsHC 360, 1 mg L^{-1}, prepared in methanol; chromatogram (c) of the mix of the synthesized AsFA 362 and AsHC 360, 1 mg L^{-1}, prepared in methanol; and overlaid chromatograms (d, e).
Gradient (0.2 mL min^{-1}) with after column dilution (0.7 mL min^{-1}, 1% nitric acid): 0–8 min, 0% B; 8–10 min, 0→100% B; 10–39 min, 100% B; 39–40 min, 100→0% B. Mobile phase A: 75 mmol L^{-1} ammonium nitrate in water (pH=9.0, 1% methanol). Columns: Thermo Scientific Dionex IonPac CG5A and Shimadzu Shim-pack Scepter C8-120 (4.6×150 mm, 3 µm). Injection volume: 10 µL.

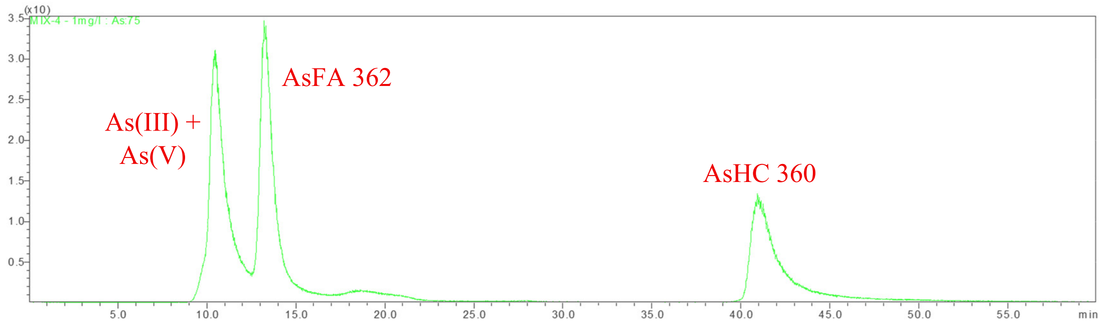

Figure S10. Chromatogram of the mix of 4 arsenic species — As(III), As(V), AsFA 362 and AsHC 360, 1 mg L^{-1}, prepared in methanol; t_{R1} = 10.452, t_{R2} = 13.223, t_{R2} = 40.923.
Gradient (0.2 mL min^{-1}) with after column dilution (0.7 mL min^{-1}, 1% nitric acid): 0–2 min, 0% B; 2–4 min, 0→100% B; 4–59 min, 100% B; 59–60 min, 100→0% B. Mobile phase A: 10 mmol L^{-1} ammonium acetate in water (pH=6.0). Columns: Thermo Scientific Dionex IonPac CG5A and Shimadzu Shim-pack Scepter C18-120 (4.6×150 mm, 3 µm). Injection volume: 10 µL.

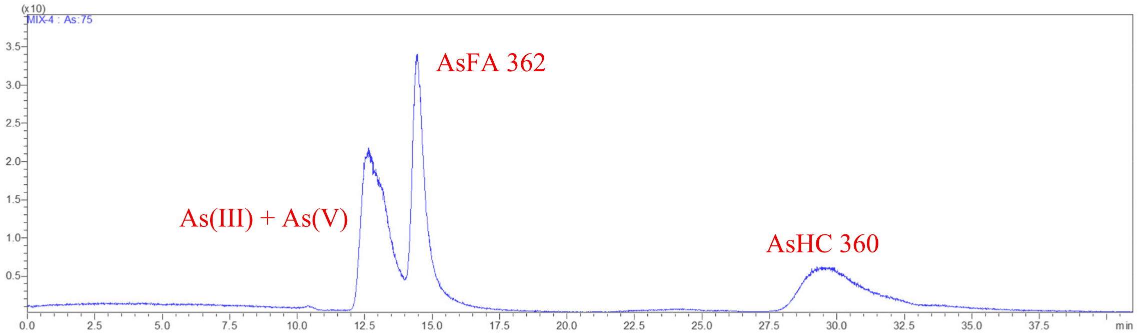

Figure S11. Chromatogram of the mix of 4 arsenic species — As(III), As(V), AsFA 362 and AsHC 360, 1 mg L^{-1}, prepared in methanol; t_{R1} = 12.651, t_{R2} = 14.453, t_{R3} = 29.416.
Gradient (0.4 mL min^{-1}) with after column dilution (0.7 mL min^{-1}, 1% nitric acid): 0–4 min, 0% B; 4–6 min, 0→100% B; 6–39 min, 100% B; 39–40 min, 100→0% B; 40–41 min, 0% B. Mobile phase A: 10 mmol L^{-1} ammonium acetate in water (pH=6.0). Columns: Thermo Scientific Dionex IonPac CG5A, Thermo Scientific Dionex IonPac AS22 and Shimadzu Shim-pack Scepter C18-120 (4.6×150 mm, 3 µm). Injection volume: 10 µL.

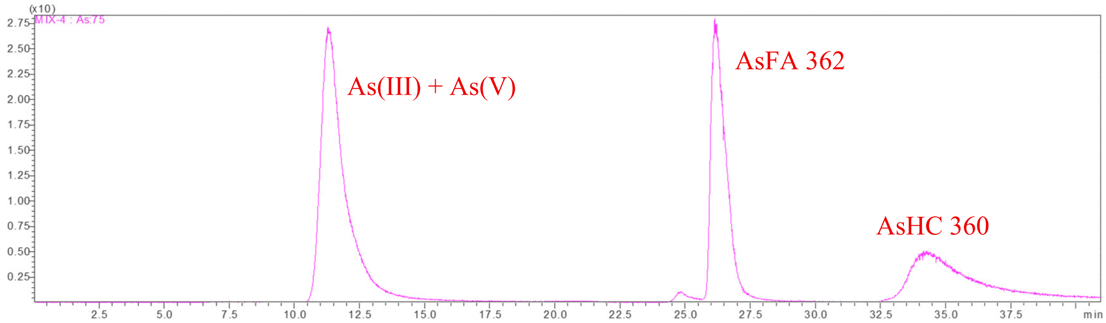

Figure S12. Chromatogram of the mix of 4 arsenic species — As(III), As(V), AsFA 362 and AsHC 360, 1 mg L^{-1}, prepared in methanol; t_{R1} = 11.279, t_{R2} = 26.139, t_{R3} = 34.250.
Gradient (0.4 mL min^{-1}) with after column dilution (0.7 mL min^{-1}, 1% nitric acid): 0–4 min, 0% B; 4–6 min, 0→100% B; 6–39 min, 100% B; 39–40 min, 100→0% B; 40–41 min, 0% B. Mobile phase A: 20 mmol L^{-1} ammonium acetate in water (pH=9.2). Columns: Thermo Scientific Dionex IonPac CG5A, Thermo Scientific Dionex IonPac AS22 and Shimadzu Shim-pack Scepter C18-120 (4.6×150 mm, 3 µm). Injection volume: 10 µL.

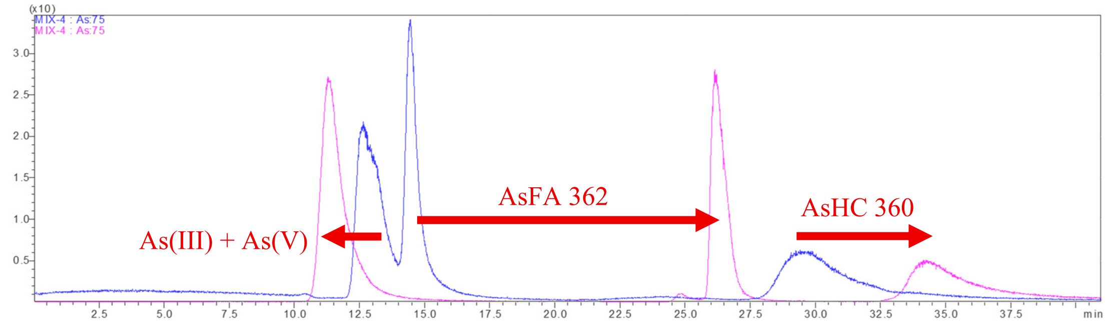

Figure S13. Overlaid chromatograms, which were presented in figures S11 and S12. *Showing how the change of the mobile phase A — from 10 mmol L^{-1} ammonium acetate in water (pH=6.0) to 20 mmol L^{-1} ammonium acetate in water (pH=9.2) — impacted the separation of the arsenic species.*

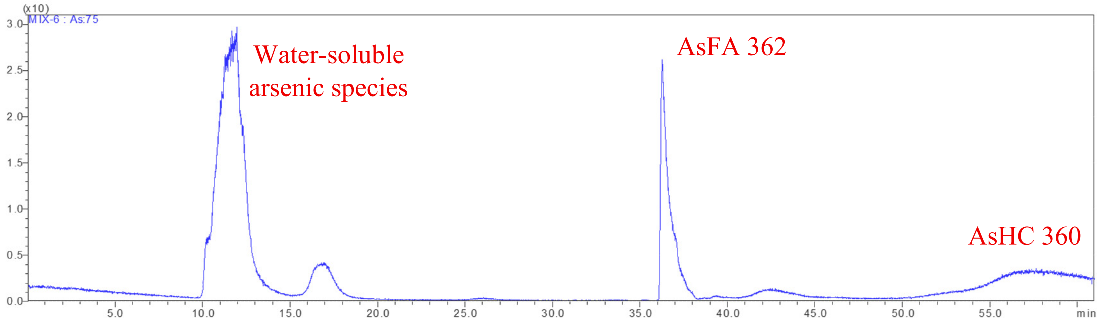

(a) t_{R1} = 11.959, t_{R2} = 16.957, t_{R3} = 36.284, t_{R4} = 57.646

Mobile phases: A — 20 mmol L^{-1} NH_4OAc in water (pH=9.2), B — 10 mmol L^{-1} NH_4OAc in methanol (pH=6.0)

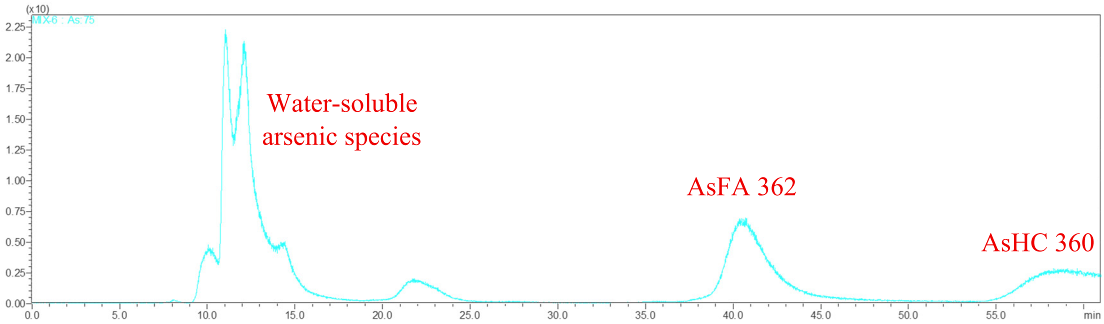

(b) t_{R1} = 10.058, t_{R2} = 11.033, t_{R3} = 12.084, t_{R4} = 14.451, t_{R5} = 21.698, t_{R6} = 40.701, t_{R7} = 59.641

Mobile phases: A — 10 mmol L^{-1} NH_4OAc in water (pH=6.0), B — 10 mmol L^{-1} NH_4OAc in methanol (pH=6.0)

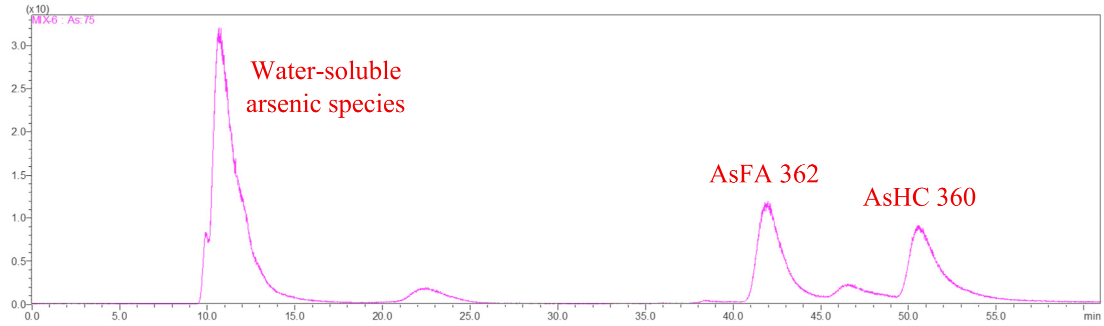

(c) t_{R1} = 9.882, t_{R2} = 10.678, t_{R3} = 22.579, t_{R4} = 41.982, t_{R5} = 46.586, t_{R6} = 50.543

Mobile phases: A — 10 mmol L^{-1} NH_4OAc in water (pH=6.0), B — 20 mmol L^{-1} NH_4OAc in methanol (pH=9.2)

Figure S14. Chromatograms (a–c) of the mix of 6 arsenic species — As(III), As(V), AsB, DMA, AsFA 362 and AsHC 360, 1 mg L^{-1}, prepared in methanol.

Gradient (0.4 mL min^{-1}) with after column dilution (0.7 mL min^{-1}, 1% nitric acid): 0–15 min, 0% B; 15–20 min, 0→100% B; 20–60 min, 100% B; 60–61 min, 100→0% B. Columns: Thermo Scientific Dionex IonPac CG5A, Thermo Scientific Dionex IonPac AS22 and Shimadzu Shim-pack Scepter C18-120 (4.6×150 mm, 3 µm). Injection volume: 10 µL.

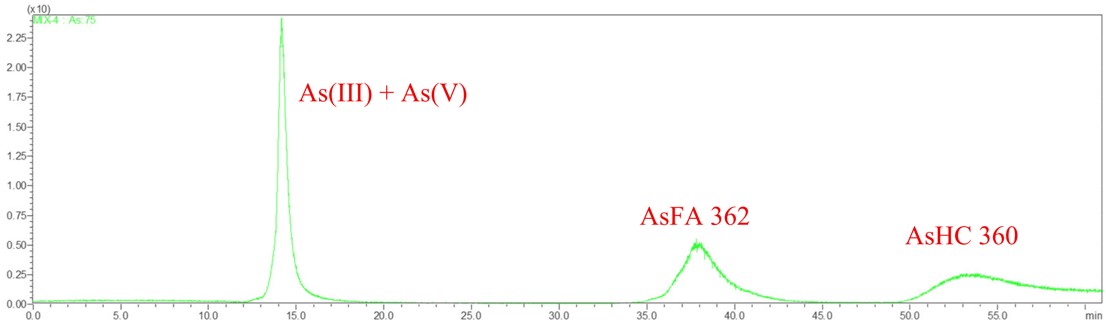

Figure S15. Chromatogram of the mix of 4 arsenic species — As(III), As(V), AsFA 362 and AsHC 360, 1 mg L^{-1}, prepared in methanol; t_{R1} = 14.164, t_{R2} = 37.825, t_{R3} = 53.725.
Gradient (0.4 mL min^{-1}) with after column dilution (0.7 mL min^{-1}, 1% nitric acid): 0–15 min, 0% B; 15–20 min, 0→100% B; 20–60 min, 100% B; 60–61 min, 100→0% B. Mobile phase A: 10 mmol L^{-1} NH_4OAc in water (pH=6.0). Mobile phase B: 10 mmol L^{-1} NH_4OAc in methanol (pH=6.0). Columns: Shimadzu Shim-pack Scepter C18-120 (4.6×150 mm, 3 μm), Thermo Scientific Dionex IonPac AS22 (4.0×250 mm, 6 μm) and Thermo Scientific Dionex IonPac CG5A. Injection volume: 10 μL.

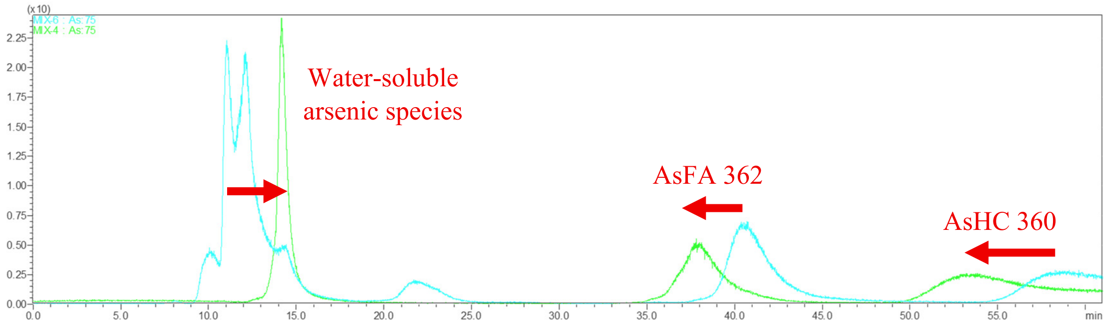

Figure S16. Overlaid chromatograms, which were presented in figures S14.b and S15. *Showing how the change of the column order — from CG5A→AS22→C18-120 to C18-120→AS22→CG5A — impacted the separation of the arsenic species.*

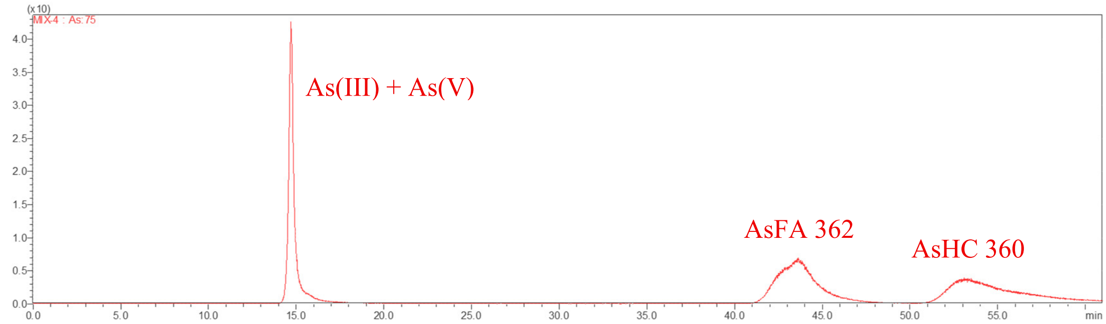

Figure S17. Chromatogram of the mix of 4 arsenic species — As(III), As(V), AsFA 362 and AsHC 360, 1 mg L^{-1}, prepared in methanol; t_{R1} = 14.705, t_{R2} = 43.606, t_{R3} = 53.073.
Gradient (0.4 mL min^{-1}) with after column dilution (0.7 mL min^{-1}, 1% nitric acid): 0–15 min, 0% B; 15–20 min, 0→100% B; 20–60 min, 100% B; 60–61 min, 100→0% B. Mobile phase A: 10 mmol L^{-1} NH_4OAc in water (pH=6.0). Mobile phase B: 10 mmol L^{-1} NH_4OAc in methanol (pH=6.0). Columns: Shimadzu Shim-pack Scepter C18-120 (4.6×150 mm, 3 μm) and Hamilton PRP-X110 (4.6×250 mm, 7 μm). Injection volume: 10 μL.

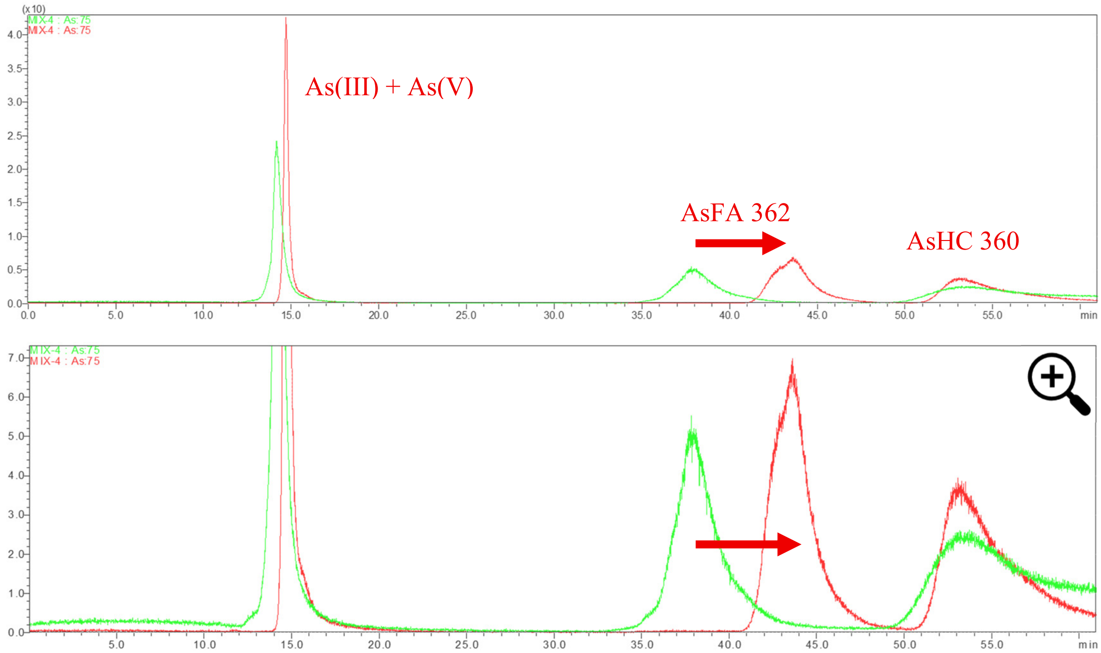

Figure S18. Overlaid chromatograms, which were presented in figures S15 and S17. *Showing how the change of the columns — from C18-120→AS22→CG5A to C18-120→PRP-X110 — impacted the separation of the arsenic species.*

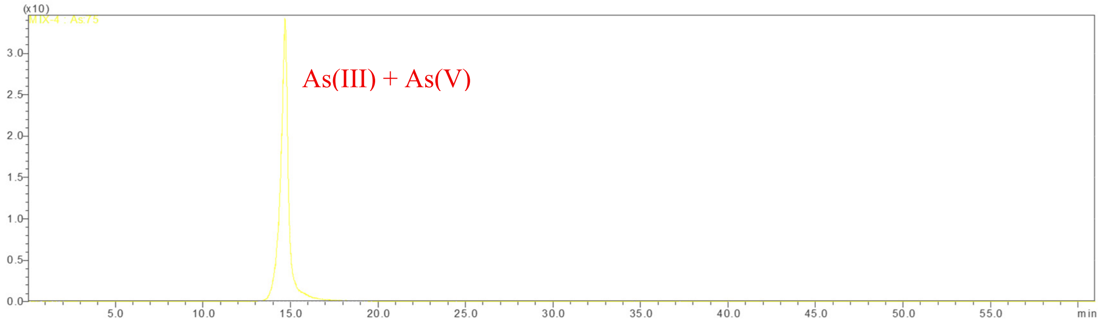

(a) t_R = 14.674

Mobile phases: A — 1 mmol L^{-1} NH_4OAc in water (pH=6.0), B — 1 mmol L^{-1} NH_4OAc in methanol (pH=6.0)

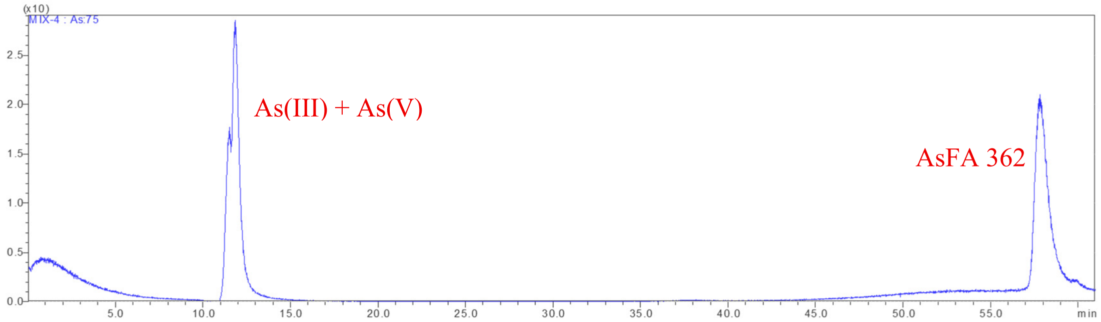

(b) t_{R1} = 0.733, t_{R2} = 11.521, t_{R3} = 11.839, t_{R4} = 57.807

Mobile phases: A — 1 mmol L^{-1} NH_4OAc in water (pH=6.0), B — 10 mmol L^{-1} NH_4OAc in methanol (pH=6.0)

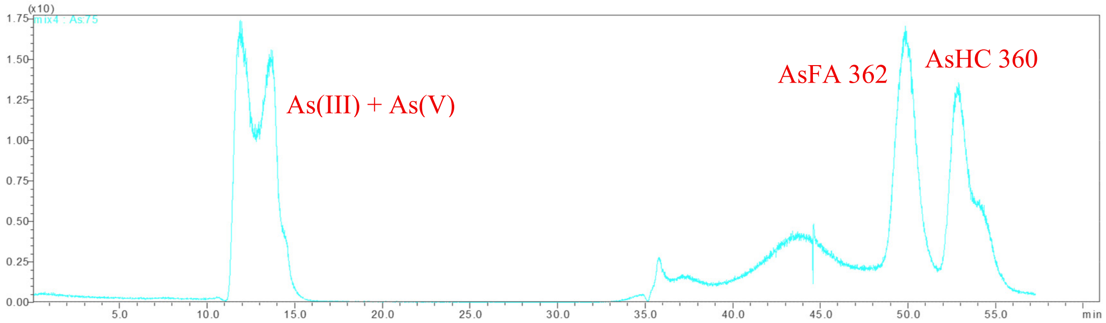

(c) t_{R1} = 11.911, t_{R2} = 13.661, t_{R3} = 34.852, t_{R4} = 35.823, t_{R5} = 37.159, t_{R6} = 44.609, t_{R7} = 49.862, t_{R8} = 52.890

Mobile phases: A — 1 mmol L^{-1} NH_4OAc in water (pH=9.2), B — 20 mmol L^{-1} NH_4OAc in methanol (pH=9.2)

Figure S19. Chromatograms (a–c) of the mix of 4 arsenic species — As(III), As(V), AsFA 362 and AsHC 360, 1 mg L^{-1}, prepared in methanol.

Gradient (0.4 mL min^{-1}) with after column dilution (0.7 mL min^{-1}, 1% nitric acid): 0–15 min, 0% B; 15–20 min, 0→100% B; 20–60 min, 100% B; 60–61 min, 100→0% B. Columns: Shimadzu Shim-pack Scepter C18-120 (4.6×150 mm, 3 µm) and Hamilton PRP-X110 (4.6×250 mm, 7 µm). Injection volume: 10 µL. *As the previous analysis was too short, the additional peaks in the chromatogram c could be of arsenolipids that were not eluted fully in the previous analysis.*

References

[9] Lai, Y.-C., et al., 2022. J Food Drug Anal. 30(4), 644–653. https://doi.org/10.38212/2224-6614.3432

[11] Sarwar, T., et al., 2021. Environ Technol Innov. 21, 101252. https://doi.org/10.1016/j.eti.2020.101252

[12] Šlejkovec, Z., et al., 2021. Food Chem. 342, 128348. https://doi.org/10.1016/j.foodchem.2020.128348

[14] Kara, S., et al., 2021. Food Chem. 356, 129706. https://doi.org/10.1016/j.foodchem.2021.129706

[18] Hackethal, C., et al., 2021. Food Chem. 346, 128913. https://doi.org/10.1016/j.foodchem.2020.128913

[19] D'Amore, T., et al., 2023. Life. 13(2), 511. https://doi.org/10.3390/life13020511

[27] Nawrocka, A., et al., 2022. Food Chem. 379, 132045. https://doi.org/10.1016/j.foodchem.2022.132045

[30] Fu, Y., et al., 2021. Environ Pollut. 280, 116958. https://doi.org/10.1016/j.envpol.2021.116958

[32] Cao, H., et al., 2022. Food Chem. 393, 133345. https://doi.org/10.1016/j.foodchem.2022.133345

[36] Camurati, J.R., et al., 2021. Food Chem. 357, 129725. https://doi.org/10.1016/j.foodchem.2021.129725

[41] Zou, H., et al., 2020. Food Chem. 327, 127033. https://doi.org/10.1016/j.foodchem.2020.127033

[45] Amin, M.H.A., et al., 2018. Food Chem Toxicol. 118, 245–251. https://doi.org/10.1016/j.fct.2018.05.019

[48] Glabonjat, R.A., et al., 2014. Anal Chem. 86(20), 10282–10287. https://doi.org/10.1021/ac502488f

[49] Glabonjat, R.A., et al., 2020. Life. 10(6), 93. https://doi.org/10.3390/life10060093

[50] Glabonjat, R.A., et al., 2021. Environ Sci Technol. 55(8), 5515–5524. https://doi.org/10.1021/acs.est.0c06901

[51] Pétursdóttir, Á.H., et al., 2019. Anal Bioanal Chem. 411(19), 4973–4985. https://doi.org/10.1007/s00216-019-01907-x

[64] Taleshi, M.S., et al., 2014. Organometallics. 33(6), 1397–1403. https://doi.org/10.1021/om4011092

[67] Stiboller, M., et al., 2019. J Anal At Spectrom. 34, 2440–2450. https://doi.org/10.1039/C9JA00249A

[68] Viczek, S.A., et al., 2016. Angew Chem Int Ed. 55(17), 5259–5262. https://doi.org/10.1002/anie.201512031

[69] Xiong, C., et al., 2022. Ecotoxicol Environ Saf. 239, 113662. https://doi.org/10.1016/j.ecoenv.2022.113662

[70] Xiong, C., et al., 2024. J Sci Food Agric. 104(11), 6957–6965. https://doi.org/10.1002/jsfa.13528

[73] Chacon-Teran, M.A., et al., 2023. Synthesis 55(24), 4091–4095. https://doi.org/10.1055/a-2122-4287

[74] Arroyo-Abad, U., et al., 2016. Eur J Lipid Sci Technol. 118, 445–452. https://doi.org/10.1002/ejlt.201400502

[78] Ghaffour, D., et al., 2025. Anal Bioanal Chem. 417, 1519–1530. https://doi.org/10.1007/s00216-025-05739-w

[79] Ignacio, S., et al., 2025. Arch Environ Contam Toxicol. 88, 253–276. https://doi.org/10.1007/s00244-025-01123-y

[80] Jakkielska, D., et al., 2024. J Hazard Mater. 471, 134364. https://doi.org/10.1016/j.jhazmat.2024.134364

[81] Lau, C., et al., 2025. J Environ Sci. 153, 302–315. https://doi.org/10.1016/j.jes.2024.12.010

[82] Lau, C., et al., 2025. J Environ Sci. 153, 289–301. https://doi.org/10.1016/j.jes.2024.12.009

[83] Navaretnam, R., et al., 2025. Environ Geochem Health. 47(10), 411. https://doi.org/10.1007/s10653-025-02723-2

[84] Qin, C., et al., 2025. Environ Sci Pollut Res. 32, 5874–5883. https://doi.org/10.1007/s11356-024-35626-3

[85] Walenta, M., et al., 2024. Anal Bioanal Chem. 416(6), 1399–1405. https://doi.org/10.1007/s00216-024-05132-z

[86] Zarić, N.M., et al., 2022. J Hazard Mater. 432, 128614. https://doi.org/10.1016/j.jhazmat.2022.128614

[87] Zhou, S., et al., 2024. Anal Sci. 40, 555–562. https://doi.org/10.1007/s44211-023-00472-9
